# International survey of parental and patient perspectives on emerging adrenaline devices for community‐based management of anaphylaxis: An EAACI task force report

**DOI:** 10.1111/pai.70507

**Published:** 2026-10-14

**Authors:** M. F. Gonzalez‐Matamala, N. Patel, D. Kahn‐Santoro, R. Knibb, G. Roberts, P. J. Turner, A. Kazie, N. Atzert, J. Bartra, K. Chetty, A. Deschildre, F. A. Dumitru, W. Feleszko, J. Gerdts, M. H. Gowland, T. T. M. Le, H. Pité, C. Ozdemir, K. Papadopoulou, M. Podestà, C. Raviolo, M. Ruano‐Zaragoza, H. O. Skjerven, B. Stoilov, L. D. Valderrama, C. Venter, C. Woehlk, G. H. Yonamine, M. Vazquez‐Ortiz, the EAACI Taskforce on Food Allergen Ingestion Risk, M. Sheehan, A. DunnGalvin, A. Alvarez‐Perea, K. Khaleva, I. Skypala, S. A. Hall, U. Nurmatov

**Affiliations:** ^1^ Centro del Alergico Santiago de Chile Chile; ^2^ Allergy Department Hospital Clínic Barcelona Barcelona Spain; ^3^ Institut d'Investigacions Biomèdiques August Pi i Sunyer (IDIBAPS) University of Barcelona Barcelona Spain; ^4^ Section of Inflammation, Repair and Development, National Heart and Lung Institute Imperial College London London UK; ^5^ Department of Paediatric Allergy Imperial College Healthcare NHS Trust London UK; ^6^ Allergy MSc Alumni, National Heart and Lung Institute Imperial College London London UK; ^7^ Institute of Health and Neurodevelopment Aston University Birmingham UK; ^8^ Faculty of Medicine University of Southampton Southampton UK; ^9^ University Hospital Southampton NHS Foundation Trust Southampton UK; ^10^ Allergiehelden Private Medical Practice Bonn Germany; ^11^ RETIC ARADyAL, RICORS de Enfermedades Inflamatorias REI (ISCIII) Madrid Spain; ^12^ Univ. Lille CHU de Lille, Service de Pneumologie et d'Allergologie Pédiatrique Lille France; ^13^ Hôpital Jeanne de Flandre Lille France; ^14^ Imperial College Healthcare NHS Trust London UK; ^15^ Department of Pediatric Pneumonology and Allergy Medical University of Warsaw Warsaw Poland; ^16^ Food Allergy Canada North York Ontario Canada; ^17^ Allergy Action St Albans UK; ^18^ Department of Dermatology/Allergology, University Medical Center Utrecht Utrecht University Utrecht The Netherlands; ^19^ Immunoallergy Department CUF Tejo Hospital Lisbon Portugal; ^20^ NOVA Medical School Universidade NOVA de Lisboa Lisbon Portugal; ^21^ Department of Pediatric Basic Sciences, Institute of Child Health Istanbul University Istanbul Türkiye; ^22^ Istanbul Faculty of Medicine, Department of Pediatrics, Division of Pediatric Allergy and Immunology Istanbul University Istanbul Türkiye; ^23^ Alder Hey Children's NHS Foundation Trust Liverpool UK; ^24^ Food Allergy Italia Padova Italy; ^25^ European Federation of Allergy and Airways Diseases Patients' Associations (EFA) Brussels Belgium; ^26^ General and Family Medicine, CAP Sant Just Desvern Barcelona Spain; ^27^ Pediatric Department of Allergy and Pulmonary Diseases and Clinical Research Unit for Children, Division of Paediatric and Adolescent Medicine, Institute of Clinical Medicine Oslo University Hospital and University of Oslo Oslo Norway; ^28^ Department of Obstetrics and Gynecology Medical University of Plovdiv Plovdiv Bulgaria; ^29^ Department of Paediatrics Royal Berkshire NHS Foundation Trust Reading UK; ^30^ Section of Allergy and Immunology, Department of Pediatrics, Children's Hospital Colorado University of Colorado School of Medicine Aurora Colorado USA; ^31^ Respiratory Research Unit, Department of Respiratory and Infectious Medicine Bispebjerg and Frederiksberg Hospital Copenhagen Denmark; ^32^ Division of Nutrition, Instituto da Criança e Do Adolescente Hospital das Clínicas da Universidade de São Paulo São Paulo Brazil

**Keywords:** adrenaline, anaphylaxis, auto‐injector, epinephrine, intranasal, patient perspectives, sublingual

## Abstract

**Introduction:**

Intranasal adrenaline has been approved in multiple countries for anaphylaxis treatment, based primarily on pharmacokinetic/pharmacodynamic comparisons to adrenaline autoinjectors (AAIs).

**Aim:**

To assess patient and caregiver perspectives regarding emerging non‐injectable adrenaline routes.

**Methods:**

Online survey of adults and caregivers of children with food allergy prescribed AAIs, with international dissemination supported by the European Academy of Allergy and Clinical Immunology (February–April 2025).

**Results:**

We analyzed 542 responses from caregivers and 224 from adults with food allergy. Effectiveness of adrenaline nasal spray (ANS) compared to AAI was rated “extremely important” by 78% of caregivers and 71% of adults. 48% of caregivers and 42% of adults were “extremely worried” that ANS may not work as well as AAI, and 50% and 44% (respectively) that it may not work well enough. Compared with AAI, respondents were less worried about ANS being painful or difficult to carry, and about fear/embarrassment around use, but more worried about side effects and knowing how to use it. Most anticipated being more comfortable using ANS than AAI, and over half anticipated earlier use during anaphylaxis. Only 17.2% of caregivers and 23.2% of adults preferred to be prescribed ANS only. There was interest in sublingual adrenaline due to portability and reduced needle‐related burden, but confidence depended on comparable speed/effectiveness and feasibility during reactions.

**Conclusions:**

Non‐injectable adrenaline is attractive to caregivers and adults, and may improve willingness to carry and use adrenaline. However, demand for evidence of effectiveness, safety, and real‐world feasibility is high, highlighting important information needs for shared decision‐making.

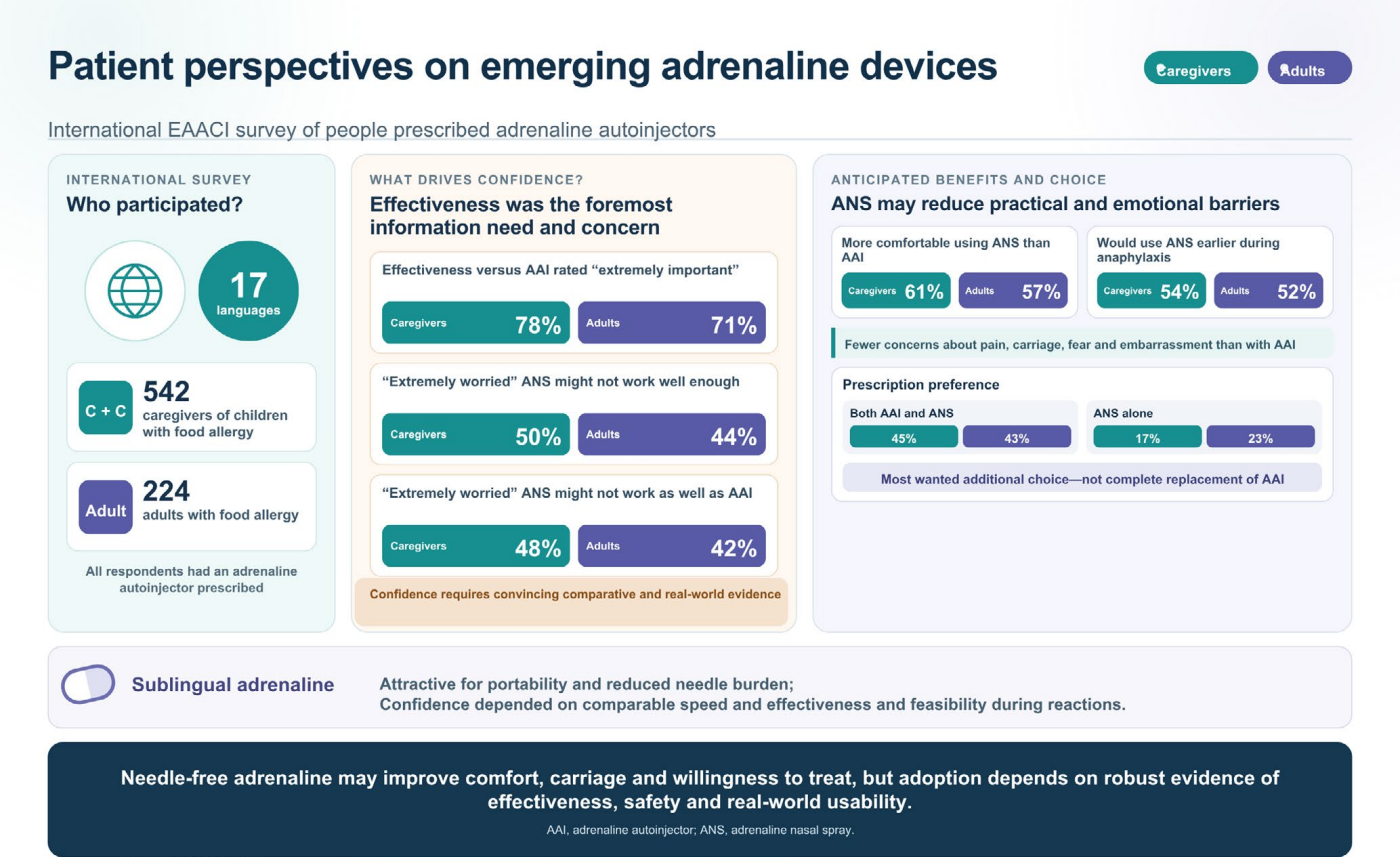

## Graphical abstract

In this international survey, caregivers and adults living with food allergy reported that they would feel more comfortable using nasal adrenaline and would use it earlier during anaphylaxis. However, effectiveness relative to autoinjectors was their foremost information need and concern, and relatively few preferred nasal adrenaline alone.

**Abbreviations**
AAI: Adrenaline autoinjector
ANS: Adrenaline nasal spray
EAACI: European Academy of Allergy and Clinical Immunology
FA: Food allergy
FAIR: Food Allergen Ingestion Risk
FAQLQ: Food Allergy Quality of Life Questionnaire
GDPR: General Data Protection Regulation
HRQoL: Health‐Related Quality of Life

**Key message**

Needle‐free adrenaline devices could reduce barriers to emergency anaphylaxis treatment by improving comfort, carriage, and willingness to act. In this international survey, caregivers of children and adults living with food allergy welcomed these options, but confidence depended on convincing evidence that new routes are effective, safe, and practical during real allergic reactions, supporting future research, counseling, and shared prescribing decisions.

## 1 BACKGROUND

Anaphylaxis is a serious allergic reaction that can progress rapidly and may cause death.^1^ A widely cited European systematic review estimated that around 1 in 300 of the general population will experience anaphylaxis at some point in their lives.^2^ Food allergy is the leading cause of anaphylaxis in children and young people.^3^ Intramuscular adrenaline is the first‐line treatment for anaphylaxis, and delays are associated with more severe outcomes including biphasic reactions and death.^4^ Current guidelines^5^ recommend that patients at risk of anaphylaxis are prescribed adrenaline autoinjectors (AAI) to facilitate rapid treatment of community‐based reactions. Fear of life‐threatening reactions, together with concerns over recognizing anaphylaxis and using adrenaline correctly, are key drivers of anxiety and reduced health‐related quality of life (HRQoL) among individuals and families at risk of anaphylaxis.^6^ Prescriptions of AAI are increasing, e.g., in the UK prescriptions increased by 336% between 1998 and 2018,^7^ imposing a significant cost burden on healthcare systems. Paradoxically, AAIs are underused even when prescribed to treat anaphylaxis.^1^, ^8^ Reasons include non‐carriage due to their size, needle‐phobia, embarrassment, fear of side effects and lack of recognition as to when treatment is justified.^9^

An adrenaline nasal spray (ANS) or sublingual preparation could address some of these concerns. EURneffy® (neffy® in USA), a single‐use ANS,^10^ was approved in the US and Europe in 2024, and the UK in 2025 for adults and children weighing ≥30 kg; UK authorisation was extended in June 2026 to the 1‐mg formulation for children aged ≥4 years weighing 15 to <30 kg.^11^, ^12^ It is also approved in Australia, Canada, China and Japan. Because randomized efficacy trials during anaphylaxis are ethically and practically difficult, regulatory approval relied primarily on pharmacokinetic and pharmacodynamic comparisons with approved injectable adrenaline products.^13^, ^14^ Subsequent prospective and real‐world clinical evidence is emerging but remains limited.^13^ An open‐label, uncontrolled study of 15 children treated for moderate reactions during oral food challenges reported that mean symptom grades generally began improving within 5 min and the median time to complete resolution was 16 min; seven children also received supportive treatments, and one later required intramuscular adrenaline for a biphasic reaction.^13^ Direct comparative‐effectiveness evidence and larger, longer‐term studies of community use remain lacking.^13^, ^14^ EURneffy represents the first major development in adrenaline devices for decades, with a view to improving patient treatment choice. Other nasal formulations and sublingual adrenaline preparations are under investigation, including AQST‐109, with phase 3 pharmacokinetic and pharmacodynamic data published.^15^

In this setting of sparse clinical efficacy data, we aimed to characterize adults' and caregivers' information needs and concerns regarding ANS; compare perceived barriers to ANS and AAIs; assess anticipated carriage, timing of use and prescription preferences; explore differences between age groups; and examine views on sublingual adrenaline. Understanding these end‐user priorities is important for guiding real‐world research, counseling, shared prescribing decisions and the safe implementation of emerging adrenaline devices.

## 2 METHODS

### 2.1 Study design and setting

This was an international, cross‐sectional online survey developed by the European Academy of Allergy and Clinical Immunology (EAACI) Task Force on Food Allergen Ingestion Risk (FAIR) to assess perspectives on the recently approved ANS and the sublingual route for anaphylaxis acute management, in the context of a broader survey assessing experiences of people living with food allergy. The survey was hosted on Qualtrics and disseminated internationally through EAACI communication channels and affiliated patient organizations and national allergy societies between February and April 2025.

### 2.2 Participants and eligibility

Two respondent groups were targeted: (1) caregivers (parents/guardians/carers) of children aged 0–18 with food allergy, and (2) adults and young people living with food allergy aged ≥16 years. Eligibility was assessed using initial screening questions within the survey. Respondents were eligible if they (or their child) had food allergy and met the age requirements. Only respondents who reported that they/their child had been prescribed AAIs were eligible to complete the ANS module questions presented in this paper. Because the survey was openly disseminated without a defined sampling frame, a conventional overall response rate could not be calculated. We therefore calculated the proportion of eligible respondents who completed the ANS module, both overall and separately before and after correction on 5/3/2025 of a routing error that had prevented some eligible respondents from accessing the ANS module. Baseline characteristics of eligible module completers and non‐completers were compared.

### 2.3 Survey development, structure, and content analyzed in this manuscript

The survey was developed by a multidisciplinary taskforce including clinicians (allergists/pediatric allergists, psychologist, dietitian, nurse) and patient representatives. The broader FAIR survey comprised (i) core questions on demographics and food allergy history, accidental reactions, preparedness/support, and HRQoL, plus (ii) optional questions addressing food‐allergen ingestion risk across settings, and (iii) optional questions on emergency adrenaline treatment, including ANS.

This manuscript reports only items relevant to AAI and non‐injectable adrenaline use and views, together with contextual demographic/clinical variables and HRQoL measures. Other questions will be reported separately. The survey instruments used for this analysis are provided in Appendix S1.

The survey was translated into 17 languages (English, French, Spanish, Portuguese, German, Italian, Greek, Turkish, Polish, Finnish, Swedish, Norwegian, Danish, Dutch, Hungarian, Bulgarian, and Romanian), and eight regional variants using forward–backward translation and cultural adaptation procedures to support linguistic accuracy and cross‐cultural validity.

Key domains analyzed were as follows:

1. Demographics and clinical characteristics.
2. Accidental reactions and AAI use within the past 3 years, and self‐reported AAI carriage frequency.
3. Perceived severity of food allergy and confidence in managing food allergy (ordinal response scales).
4. HRQoL using the Food Allergy Quality of Life Questionnaire (FAQLQ)‐Adult Form items in the adult survey,^16^ and an adapted caregiver version developed within this Task force initiative (manuscript near submission).
5. Information needs regarding ANS, rated on a 0–4 importance scale (0: not at all to 4: extremely important).
6. Concerns and perceived barriers for AAI and ANS use, measured on a 0–4 worry scale (0: not at all to 4: extremely worried).
7. Anticipated use and preferences regarding ANS, including anticipated ANS carriage frequency, comfort compared with AAI, perceived impact on readiness to treat, and prescription preference.
8. Perspectives on sublingual adrenaline, additional information needs and worries on ANS and AAI as free‐text questions.

### 2.4 Data handling, security, and ethics

Participation was voluntary and anonymous. An electronic information/consent statement was included. No identifiable personal data were collected. Survey data were stored in GDPR‐compliant systems and access was restricted to the study team. The survey protocol received institutional approval by the Research Ethics Committee at Imperial College London, UK (reference: 6871034).

### 2.5 Statistical analysis plan

Analyses were conducted separately for caregivers and adults. Primary outcomes were ANS information needs, concerns/perceived barriers, and anticipated use and prescription preferences. Descriptive statistics were used to summarize responses, reported as number (%) and mean (SD) or median (IQR), as appropriate. HRQoL items, measured on a 0–6 Likert scale (0 = not at all, 6 = extremely), were analyzed descriptively and presented as mean (SD) and distributions of responses. Analyses used available‐case denominators per item; no imputation was performed.

For between‐group comparisons (caregivers vs. adults), categorical distributions were compared using *χ* ^2^ tests (or Fisher's exact test, as appropriate). For within‐respondent comparisons of concerns/barriers for AAI vs. the corresponding ANS item (paired ordinal outcomes on the 0–4 worry scale) and carriage, the primary comparison used the Wilcoxon signed‐rank test.

To explore age‐related differences, post hoc subgroup analyses were conducted for the key outcomes. Caregivers' information needs and AAI‐ and ANS‐related concerns were compared across child age groups (0–2, 3–10, and 11–18 years) using Kruskal–Wallis tests; a significant omnibus test was followed by Bonferroni‐adjusted pairwise comparisons. In the patient analysis, respondents aged 16–25 years were compared with those aged >25 years across information needs, concerns, anticipated ANS use, and device and prescription preferences, using Mann–Whitney *U*, Pearson's *χ* ^2^ or Fisher–Freeman–Halton exact tests, as appropriate. In post hoc exploratory analyses, Spearman's rank correlations were used to examine associations between mean food‐allergy‐related HRQoL scores and the three effectiveness‐related concerns, separately for caregivers and adults. Pairwise complete observations were used, and *p*‐values were adjusted using the Holm method.

Statistical significance was set at *p* < .05 (two‐sided). No multiplicity adjustment was applied to the original exploratory item‐level comparisons; adjustments were applied to the additional secondary and post hoc analyses as above. IBM SPSS Statistics for Windows, version 30.0 (IBM Corp., Armonk, NY, USA) was used.

Free‐text responses were analyzed using thematic analysis following the Braun and Clarke approach^17^ (see Appendix S1).

## 3 RESULTS

### 3.1 Demographics and food allergy characteristics

Of 1890 caregiver respondents of the broader FAIR survey, 1528 (80.8%) were eligible for the supplementary ANS module and 542/1528 (35.5%) completed it. Completion increased from 107/1034 (10.3%) before correction of the routing error to 435/494 (88.1%) afterwards. Among 720 adult respondents to the broader FAIR survey, 512 (71.1%) were eligible and 224/512 (43.8%) completed the ANS module; completion increased from 78/361 (21.6%) before correction to 146/151 (96.7%) afterwards. In comparisons between eligible ANS module completers and non‐completers, country distribution was the only baseline characteristic that differed following Holm adjustment in both caregivers (*χ* ^2^(17) = 279.36; *n* = 1483) and adults (*χ* ^2^(9) = 61.59; *n* = 487; both adjusted *p* < .001); no other comparison remained significant (data not shown).

Respondent demographics are summarized in Table 1. Most respondents were White/Caucasian, female and held a university degree. Regarding country of residence, the most common among caregivers were Portugal (15.9%), Germany (12%), Canada (8.5%), United Kingdom (8.1%), France (7.4%) and Spain (6.6%). Among adults, the most common countries were Canada (16.4%), United Kingdom (14.2%), Australia (11%), Portugal (10%), Spain (9.1%) and Germany and Chile (6.8%).

**TABLE 1:** Participant demographics.

Respondents demographics		
	Caregivers (*n* = 542)	Adults (*n* = 224)
Relationship with child with Food allergy, *n* (%)		
Mother	517 (95.4)	NA
Father	20 (3.7)	NA
Other	4 (0.7)	NA
Respondent's age (years), mean ± SD	41.3 ± 6.2	39.3 ± 14.3
Child's age (years), mean ± SD	8.2 ± 4.6	NA
Education level, *n* (%)		
Primary school	2 (0.4)	2 (0.9)
Secondary school	27 (5)	29 (13.2)
Vocational or similar	65 (12)	32 (14.5)
University degree	397 (73.2)	129 (58.9)
Other	49 (9)	27 (12.5)
Missing	2 (0.4)	5 (2.2)
Ethnicity, *n* (%)		
White/Caucasian	440 (81.2)	186 (91.2)
Black/African American	0 (0)	1 (0.5)
American Indian/Native American	0 (0)	1 (0.5)
Asian	14 (2.6)	4 (2)
Mixed	23 (4.2)	9 (4.4)
Other	11 (2)	3 (1.5)
Prefer not to say	4 (0.7)	0 (0)
Missing	50 (9.2)	20 (8.9)
Gender, *n* (%)		
Male	23 (4.2)	24 (10.9)
Female	514 (94.8)	195 (88.5)
Non binary	0 (0)	0 (0)
Prefer not to say	0 (0)	1 (0.5)
Missing	5 (0.9)	4 (1.8)
Country, *n* (%)		
Portugal	86 (15.9)	22 (10)
Germany	65 (12)	15 (6.8)
Canada	46 (8.5)	36 (16.4)
United Kingdom	44 (8.1)	31 (14.2)
Spain	36 (6.6)	20 (9.1)
France	40 (7.4)	9 (4.1)
Greece	35 (6.5)	2 (0.9)
Australia	31 (5.7)	24 (11)
Romania	30 (5.5)	4 (1.8)
Italy	23 (4.2)	13 (5.9)
United States of America	20 (3.7)	9 (4.1)
Chile	15 (2.8)	15 (6.8)
Switzerland	14 (2.6)	3 (1.4)
Netherlands	7 (1.3)	4 (1.8)
Mexico/Hungary	6 (1.1)	0 (0)
Austria/Belgium/Finland/Ireland/Turkey	4 (0.7)	0 (0)
South Africa/Serbia/Poland/Oman/New Zealand/Honduras/Denmark	1 (0.2)	0 (0)
Argentina/Belgium/Brazil/Denmark/Hong Kong/Mexico/Thailand	0 (0)	1 (0.5)
Hungary	0 (0)	2 (1)
Switzerland/Finland	0 (0)	3 (1.4)
Missing	6 (1.1)	5 (2.2)

Details regarding the food allergy diagnosis and previous reactions are reported in Table 2. Over two‐thirds of respondents reported a prior anaphylaxis reaction. Regarding perceived severity, most respondents considered the food allergy to be severe (82.0% of caregivers; 80.9% of adults), whilst only 1.3% of caregivers and 0.9% of adults considered it mild. Regarding confidence in managing food allergy, 40.8% of caregivers and only 11.4% of adults were extremely confident. HRQoL scores (range 0–6) had a mean of 4.8 (SD 1) in caregivers and 4.7 (SD 1) in adults (Figure S1).

**TABLE 2:** Clinical characteristics of food allergy in adult participants and caregivers' children.

Clinical characteristics relating to food allergy in adult respondents and caregivers' child		
	Caregivers (*n* = 542)	Adults (*n* = 224)
Age at first food allergy (FA) diagnosis, *n* (%)		
0	332 (61.3)	33 (14.7)
1–5	181 (33.4)	52 (23.2)
6–10	20 (3.7)	15 (6.7)
11–18	4 (0.7)	26 (11.6)
>18	0 (0)	81 (36.2)
Missing	5 (0.9)	17 (7.6)
FA diagnosis made by healthcare professional, *n* (%)		
Yes	523 (96.5)	214 (95.5)
No	15 (2.8)	4 (1.8)
Not sure	0 (0)	2 (0.9)
Missing	4 (0.7)	4 (1.8)
History of unexpected reaction within 2 h of eating, *n* (%)		
Yes	504 (93)	205 (91.5)
No	25 (4.6)	13 (5.8)
Not sure	7 (1.3)	2 (0.9)
Missing	6 (1.1)	4 (1.8)
History of reaction involving: *n* (%)		
Airway	315 (71.8)	174 (86.6)
Breathing	311 (70.8)	157 (78.1)
Consciousness	168 (38.3)	23 (11.4)
Missing	103 (19)	23 (10.3)
AAI currently prescribed, *n* (%)		
Yes	527 (97.2)	202 (90.2)
No	15 (2.8)	18 (8.0)
Not sure	0 (0)	0 (0)
Missing	0 (0)	4 (1.8)
Severity of FA perception, *n* (%)		
Mild	7 (1.3)	2 (0.9)
Moderate	71 (13.1)	33 (14.7)
Severe	442 (81.5)	178 (79.5)
Not sure	19 (3.5)	7 (3.1)
Missing	3 (0.6)	4 (1.8)
Number of food allergies, mean ± SD	4.6 ± 2.1	3.53 ± 2.8
Food allergy triggers, *n* (%)		
Cow's milk	190 (35.1)	35 (15.6)
Egg	206 (38)	34 (15.2)
Peanut	292 (53.9)	106 (47.3)
Tree nuts	334 (61.6)	134 (59.8)
Sesame	100 (18.5)	30 (13.4)
Mustard	30 (5.5)	17 (7.6)
Other sedes	42 (7.7)	34 (15.2)
Fish	53 (9.8)	21 (9.4)
Shellfish	39 (7.2)	59 (26.3)
Soya	43 (7.9)	29 (12.9)
Lupin	13 (2.4)	25 (11.2)
Other legumes	47 (8.7)	26 (11.6)
Wheat	42 (7.7)	24 (10.7)
Other grains	8 (1.5)	17 (7.6)
Vegetables	20 (3.7)	37 (16.5)
Fruits	70 (12.9)	74 (33.0)
Meat/chicken	13 (2.4)	11 (4.9)
Food additives	6 (1.1)	26 (11.6)
Other	161 (29.7)	37 (16.5)
Allergic reaction within last 3 years, *n* (%)		
Yes	362 (66.8)	150 (67.0)
No	175 (32.3)	69 (30.8)
Not sure	5 (0.9)	5 (2.2)
Missing	0 (0)	0 (0)
Number of reactions within last 3 years, mean ± SD	2.27 ± 1.2	2.18 ± 1.09
History of AAI administration for reactions within last 3 years, *n* (%)		
Yes	134 (36.5)	68 (43.9)
No	219 (59.7)	79 (50.1)
Not sure	14 (3.8)	8 (5.2)
Confidence in managing FA, *n* (%)		
Not at all confident	3 (0.6)	30 (13.4)
A little confident	28 (5.2)	15 (6.7)
Somewhat confident	72 (13.3)	14 (6.3)
Very confident	176 (32.5)	42 (18.8)
Extremely confident	192 (35.4)	24 (10.7)
Don't know	0 (0)	38 (17)
Missing	71 (13.1)	61 (27.2)
Quality of life (FAQLQ^a^) score (0–6), mean ± SD	4.77 ± 0.98	4.7 ± 1.02

*Note*: Values are *n* (%) unless otherwise stated; percentages use the full group denominators except for reaction features (valid *n* = 439 caregivers and 201 adults) and AAI administration within the previous 3 years (*n* = 367 and 155, respectively). Multiple responses were permitted for reaction features and food‐allergy triggers.

Abbreviations: AAI, adrenaline autoinjector; FA, food allergy; FAQLQ, Food Allergy Quality of Life Questionnaire.

^a^ Quality of life was measured using the FAQLQ‐Adult Form in adults^16^ and an adapted caregiver version developed within this Task Force initiative.

### 3.2 Perceived importance of information needs regarding ANS

Rankings of information needs relating to the ANS by importance for decision‐making were broadly similar in caregivers and adults (Figure 1). The highest‐ranked information need was effectiveness of ANS compared to AAI (caregivers: 77.5% extremely, 7.6% very important; adults: 71.0% extremely, 12.9% very). ANS effectiveness in anaphylaxis/severe reactions was the next most important information need (caregivers: 76.4%, 7.7%; adults: 75.4%, 10.3%). Other information needs included how ANS works (ranked third), the impact of nasal problems or treatments on ANS effectiveness (ranked fourth), and side‐effect profile (ranked fifth).

**FIGURE 1:**
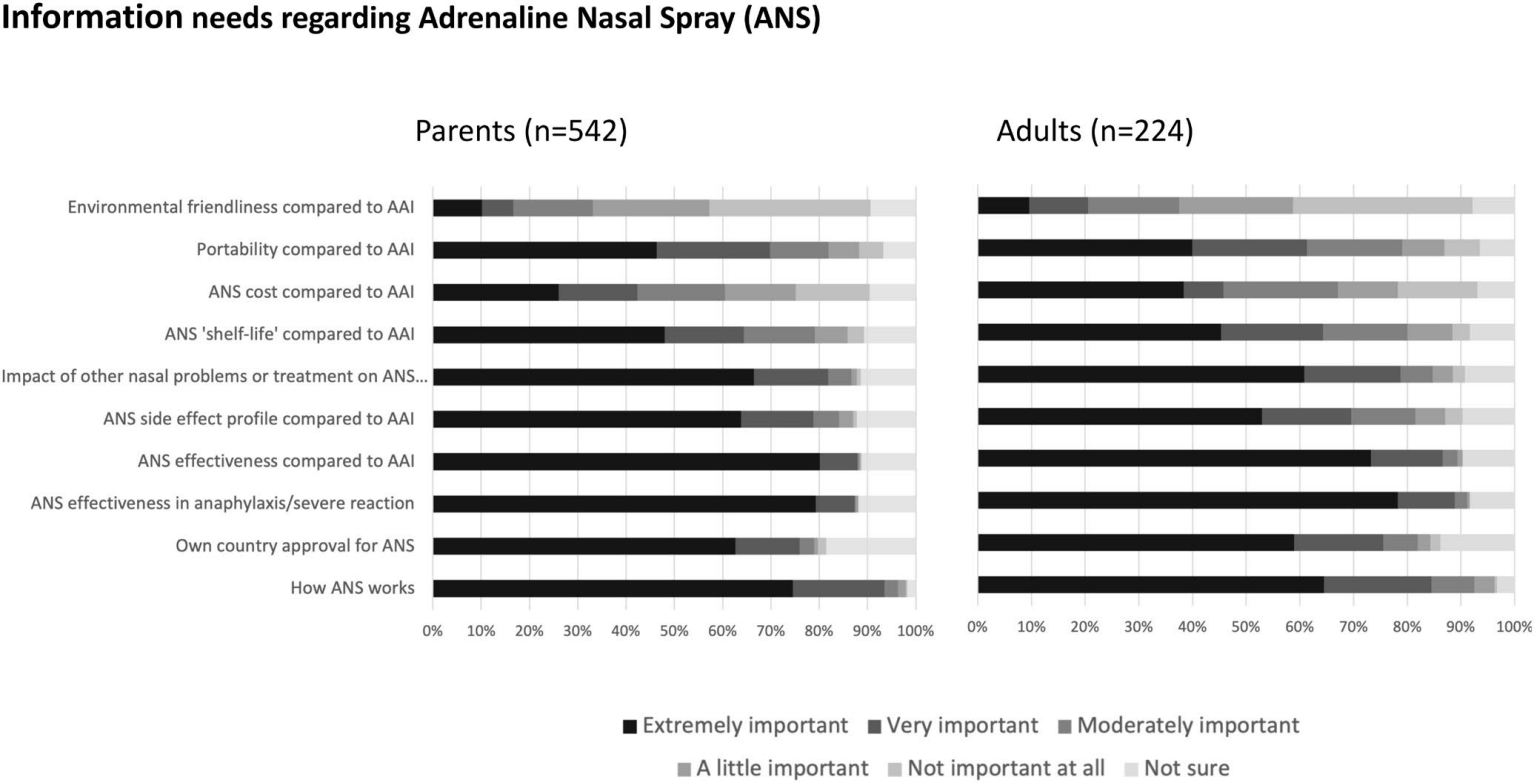
Information needs regarding Adrenaline Nasal Spray (ANS) in adults with food allergy and caregivers of children with food allergy. Group comparison caregivers vs. adults done using *χ* ^2^ test. *p value <.05.

### 3.3 Concerns and perceived barriers to ANS and AAI use

#### 3.3.1 Caregivers

Concerns regarding ANS and AAI (0–4 ‘worry scale’) in caregivers are shown in Figure 2A,B. The highest levels of worry related to perceived effectiveness, with 70.6% reporting being extremely/very worried that ANS might not work well enough (50.4% extremely, 20.2% very) and 64.2% reporting being extremely/very worried about the ANS not working as well as the AAI (48% extremely, 16.2% very). High levels of worry were also common for other people knowing how and when to use ANS (54.4%; 31.2% extremely, 23.2% very). Knowledge‐related concerns were reported by around one‐third of caregivers (when to use ANS, 32.7%; how to use ANS, 31.6%), and ANS having other side effects was rated as very/extremely worrying by 30.1%. Emotional and practical barriers were less frequently raised for ANS, including fear (parent 8.2%; child 14.0%) or embarrassment to use it (parent 2.4%; child 7.1%), and ANS not being easy/practical to carry (13.9%). In paired comparisons with AAI ratings (Wilcoxon signed‐rank, see Table S1 for detailed statistics), caregivers reported greater worry for ANS not working well enough, causing side effects and knowing how to use, whereas the worry was lower for ANS compared to AAI for pain, ease/practicality of carrying and child embarrassment, caregiver and child fear to use the ANS (*p* < .001 for all comparisons mentioned).

**FIGURE 2:**
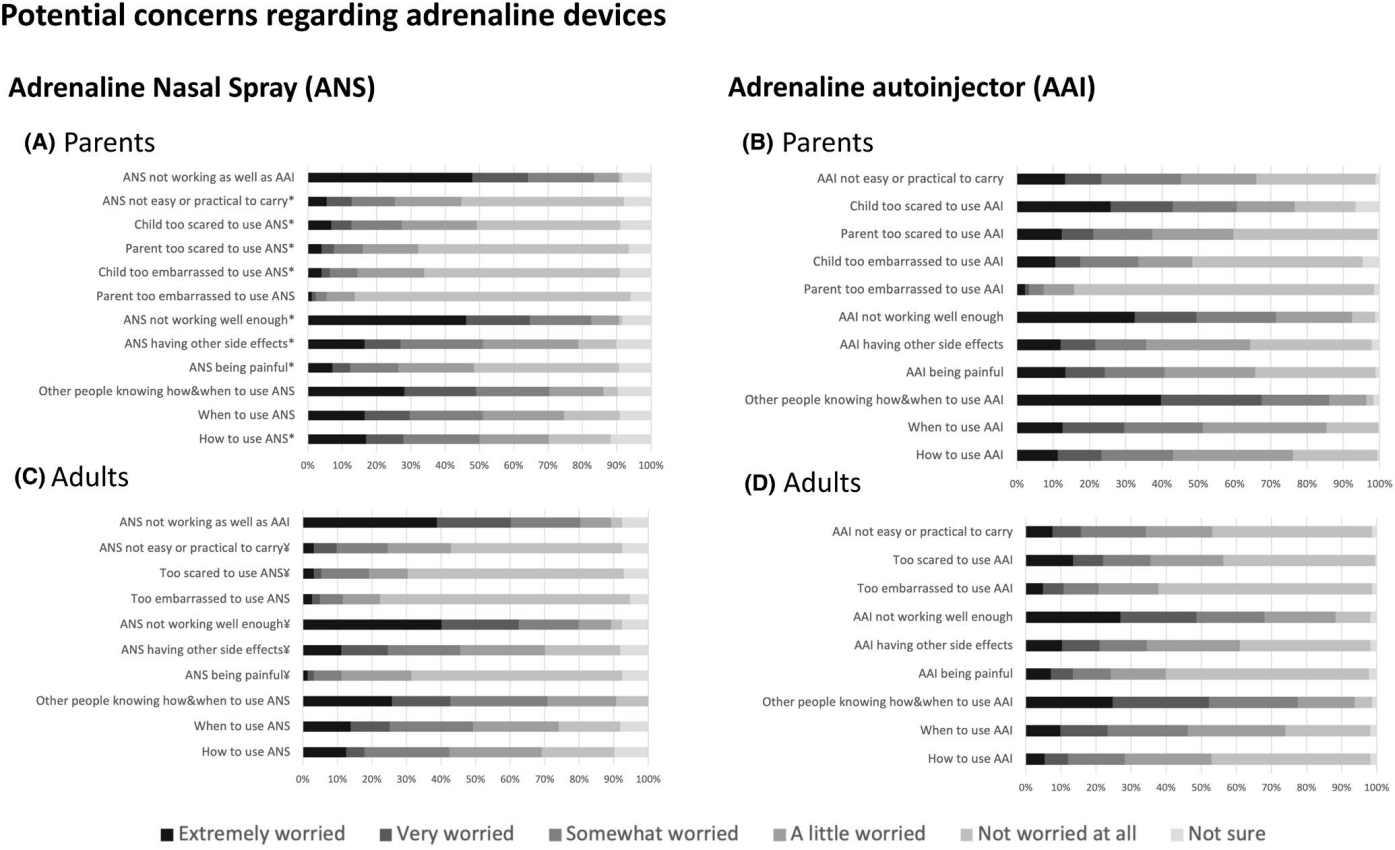
Potential concerns regarding Adrenaline Nasal Spray (ANS) and adrenaline autoinjector (AAI) in adults with food allergy and caregivers of children with food allergy. Comparison ANS vs. AAI in caregivers, and in adults done using Wilcoxon signed‐ranked test (*p value <.05 in caregivers comparison; ¥ *p* value <.05 in adults comparison).

#### 3.3.2 Adults

In adults, the overall pattern was similar to caregivers (Figure 2C,D), with the highest worry again relating to perceived ANS effectiveness: 67.6% were very/extremely worried that ANS might not work well enough (43.5% extremely, 24.2% very), and 65.2% were very/extremely worried that ANS may not work as well as an AAI (42.0% extremely, 23.2% very). Worry that other people may not know how and when to use ANS was also common (25.7% extremely, 17.0% very). Intermediate levels of worry were reported for when to use ANS (27.3% very/extremely), ANS having other side effects (26.7%), and how to use ANS (19.8%), whereas concerns were lowest for ANS being painful (3.4% very/extremely), being too embarrassed to use ANS (5.2%), being too scared to use ANS (5.8%), and ANS not being easy/practical to carry (10.6%). In paired comparisons with AAI ratings (Wilcoxon signed‐rank, see Table S2 for detailed statistics), adults similarly reported greater worry for ANS not working well enough (*p* < .001), causing other side effects (*p* < .001) and knowing how to use (*p* < .001), and lower worry for ANS than AAI for pain (*p <* .001), being too scared to use (*p* < .001), and ease/practicality of carrying (*p* = .023).

#### 3.3.3 Comparison caregivers vs. adults

Comparing concerns in caregivers of children with food allergies to concerns in food‐allergic adults (*χ* ^2^ tests, see Tables S3 and S4 for detailed statistics), caregivers more frequently reported being very/extremely worried about how to use (23.4% vs. 12.3%; *p* < .001), when to use (29.7% vs. 23.7%; *p* = .009), other people knowing how/when to use (68.6% vs. 53.0%; *p* < .001), AAI being painful (24.5% vs. 13.8%; *p* < .001), and AAI not being easy/practical to carry (23.6% vs. 16.0%; *p* = .012). Adults were more likely to report embarrassment over using an AAI as a concern (3.3% vs. 10.7%; *p* < .001). For ANS, caregivers more frequently reported being very/extremely worried about how to use (31.6% vs. 19.8%; *p* = .023), other people knowing how/when to use (54.4% vs. 42.7%; *p* = .029), ANS being painful (13.6% vs. 3.4%; *p* < .001), and ANS having other side effects (30.1% vs. 26.7%; *p* = .001).

### 3.4 Associations between HRQoL and effectiveness‐related concerns

Greater impairment in food‐allergy‐related HRQoL was associated with greater effectiveness‐related concerns in both respondent groups. Among caregivers, greater impairment in their own HRQoL was associated with greater concern that AAI and ANS might not work well enough (Spearman's *ρ* = .444; *n* = 532 and *ρ* = .366; *n* = 480, respectively), and that ANS might not work as well as AAI (*ρ* = .379; *n* = 481). Corresponding associations were observed among adults with food allergy (*ρ* = .420; *n* = 218; *ρ* = .359; *n* = 207; and *ρ* = .303; *n* = 207, respectively). All associations remained significant following Holm adjustment for six tests (all adjusted *p* < .001).

### 3.5 Anticipated use and preferences for an ANS

Table 3 summarizes responses on anticipated carriage, comfort and readiness to use, and prescription preferences for an ANS. Most respondents anticipated being more comfortable using ANS than AAI (caregivers: 61.4%; adults: 56.7%), and caregivers commonly anticipated their child would be much more comfortable using ANS (57.9%) than AAI. Over half of respondents indicated they would use ANS earlier than AAI during anaphylaxis (caregivers: 54.3%; adults: 52.2%), and most reported they would always carry ANS (caregivers: 81.9%; adults: 88.2%), higher rates compared to the reported AAI carriage rates (*p* < .001 for caregivers, *p* < .001 for adults, Wilcoxon signed‐rank test). With regard to prescription preferences, the most preferred option would be carrying both AAI and ANS (44.8% of caregivers and 42.9% of adults); only 17.2% of caregivers and 23.2% of adults would like ANS only. Full *χ* ^2^ test statistics for between‐group comparisons are provided in Table S5.

**TABLE 3:** Anticipated carriage, use and preferences for Adrenaline Nasal Spray (ANS) in adults with food allergy and caregivers of children with food allergy.

Anticipated carriage, use and preferences for adrenaline nasal spray (ANS)			
	Caregivers (*n* = 542)	Adults (*n* = 224)	*p* value (caregiver vs adult), *χ* ^2^ test
Anticipated ANS carriage, *n* (%)			
Always	417 (81.9)	194 (88.2)	.005
Most of the time	86 (16.9)	19 (8.6)	
About half the time	3 (0.6)	4 (1.8)	
Occasionally	1 (0.2)	3 (1.4)	
Never	2 (0.4)	0 (0.0)	
Don't know	0 (0.0)	0 (0.0)	
Comfort using ANS in future compared with AAI, *n* (%)			
Much less comfortable	26 (5.0)	19 (8.5)	<.001
A little less comfortable	59 (11.4)	9 (4.0)	
As comfortable	64 (12.4)	53 (23.7)	
A little more comfortable	79 (15.3)	29 (12.9)	
Much more comfortable	239 (46.1)	98 (43.8)	
Not sure	51 (9.8)	16 (7.1)	
Child's comfort using ANS in future compared with AAI, *n* (%)			
Much less comfortable	17 (3.3)	NA	NA
A little less comfortable	22 (4.3)	NA	
As comfortable	42 (8.2)	NA	
A little more comfortable	82 (15.9)	NA	
Much more comfortable	298 (57.9)	NA	
Not sure	54 (10.5)	NA	
Impact of device type on readiness to use adrenaline			
No—device type would not affect timing	192 (37.0)	83 (37.1)	.034
Yes—would use ANS earlier than AAI	282 (54.3)	117 (52.2)	
Yes—would wait longer to use ANS vs. AAI	3 (0.6)	6 (2.7)	
No—would wait for medical help	0 (0.0)	2 (0.9)	
Not sure	37 (7.1)	14 (6.3)	
Other	5 (1.0)	2 (0.9)	
Impact of device type on child's readiness to use adrenaline	Caregivers (child) *n* (%)		NA
No—device type would not affect timing	49 (9.5)	NA	
Yes—ANS would be used earlier	220 (42.6)	NA	
Yes—would wait longer to use ANS	0 (0.0)	NA	
No—would wait for medical help	2 (0.4)	NA	
Not sure	39 (7.6)	NA	
Other	5 (1.0)	NA	
My child is too young	201 (39.0)	NA	
Prescription preference if offered ANS by the healthcare team			
Prefer both AAI and ANS	231 (44.8)	96 (42.9)	<.001
Prefer AAI only	14 (2.7)	10 (4.5)	
Prefer AAI only, but would consider ANS when more information is available	144 (27.9)	55 (24.6)	
Prefer ANS only	89 (17.2)	52 (23.2)	
Not sure	26 (5.0)	8 (3.6)	
Other	12 (2.3)	3 (1.3)	

*Note*: Group comparison caregivers vs. adults done using *χ* ^2^ test.

Abbreviations: AAI, adrenaline autoinjector; ANS, Adrenaline nasal spray.

Additional perspectives from free‐text responses are reported in Tables S6–S8 and Appendix S1.

### 3.6 Age subgroup analyses

Caregivers' information needs and AAI‐ and ANS‐related concerns were compared across child age groups: 0–2 years (*n* = 49), 3–10 years (*n* = 319), and 11–18 years (*n* = 131). Following Holm adjustment across 33 omnibus item‐level comparisons, an age‐group difference was identified only for concern that the child might be too embarrassed to use an AAI (Kruskal–Wallis H(2) = 18.81; raw *p* < .001; adjusted *p* = .003). Among caregivers providing a valid response, the proportion reporting any worry increased across the three age groups from 14/46 (30.4%) to 146/297 (49.2%) and 85/128 (66.4%). Bonferroni‐adjusted pairwise comparisons showed greater concern among caregivers of children aged 11–18 years than 0–2 years (adjusted *p* < .001) and 3–10 years (adjusted *p* = .001); the two younger groups did not differ (adjusted *p* = .202). No other comparison remained significant following Holm adjustment (Table S9).

Among patient respondents, comparisons between those aged 16–25 years (*n* = 41) and >25 years (*n* = 177) identified no differences in information needs, AAI‐ or ANS‐related concerns, anticipated ANS carriage or use, or device and prescription preferences following Holm adjustment across 33 comparisons (all adjusted *p* > .05) (Table S10).

### 3.7 Perspectives on sublingual adrenaline

Responses for the relevant free‐text question were obtained from 133 adults and 304 caregivers. Reflexive thematic analysis identified five themes (Table 4): (1) ‘Reduced burden and acceptability’ captured strong interest in a needle‐free tablet, perceived as more portable, discreet, less intimidating, and easier to use than AAI; (2) ‘Confidence conditional on evidence of speed and effectiveness’ reflected that willingness to use a tablet depended on proof that it works as quickly and effectively as existing devices; (3) ‘Feasibility of sublingual dosing during allergic reactions and safety concerns’ described worries that vomiting, coughing, airway compromise, panic, or loss of consciousness could make it difficult or unsafe to keep a tablet under the tongue for the required time; (4) ‘Preference for choice and layered safety’ showed that many participants viewed the tablet as an additional option rather than a replacement; (5) ‘Acceptability constraints’ captured more specific barriers, including palatability, sensory aversion, excipients, misuse, and—particularly among caregivers—child age, cooperation, and concern that a tablet might be lost or harder for others to locate in an emergency. Overall, caregiver and adult perspectives were closely aligned, although caregivers placed greater emphasis on child cooperation and practical administration by others.

**TABLE 4:** Perspectives on sublingual adrenaline in adults with food allergy and caregivers of children with food allergy. Reflexive thematic analysis theme summary using Braun and Clark^17^ approach.

Theme	Adults: summary and illustrative quotes. *N* = 133	Caregivers: summary and illustrative quotes. *N* = 304
Reduced burden and acceptability (portable, discreet, less intimidating, easier to use)	A tablet was seen as simpler, easier to carry and use independently and by others, more discreet/less “visible,” and possibly cheaper, potentially reducing day‐to‐day burden and increasing willingness to use adrenaline. “I would definitely prefer the tablet, due to ease of carrying it!!!” “Would solve any ‘visual’ stressors or embarrassment and it would be so much easier to transport around.” “It would be wonderful—especially if… the price was lower than what currently exists on the market.”	Tablet viewed as less scary/traumatic than injection, discreet, easier to carry/store and easier to use by others (grandparents, school staff), potentially improving day‐to‐day readiness. “That would greatly reduce stress for the child.” “I think this would be preferable over a nose spray or an autoinjector. My son would feel happiest using this too” “Perfect! A tablet would be easier to transport and store.” “For grandparents, school staff and us (parents), I think administration would be faster (with tablet).”
Confidence is conditional on evidence of speed and effectiveness	Many were open to the tablet if it was shown to be as fast and effective as existing options; requests for evidence/information were common. “Everything would depend on effectiveness; if it's as effective as the autoinjector, any solution is good.” “I would be happy to try this once testing has taken place.” “There would need to be sufficient studies guaranteeing their effectiveness during anaphylactic shock.” “A minute is quite a long time, I think, compared with how quickly the spray or pen works.”	Many would consider it if proven comparable to injectors; speed/time‐to‐effect was repeatedly flagged. “If it was proven as effective as adrenaline pens I would be happy to use the tablet.” “Until real‐world data indicates as‐good‐as or better‐than effectiveness of the current injector, we would carry at least 2 doses of both.” “The real important question is which is most effective and safest.” “I would be worried about how long the pill takes to work.”
Feasibility of sublingual dosing during allergic reactions and safety concerns	Respondents worried that during anaphylaxis (panic, breathing difficulty, vomiting/swelling) it may be hard or impossible to keep a tablet in place for long enough, risking delay or failure. Some questioned whether “1 min” is too long. “I'm concerned I wouldn't keep it under my tongue properly during a reaction.” “I would expel it because of the cough” “NO way. In one minute I would have lost concentration and be unable to reliably hold it there while gasping for air.” “1 min is too long… my body deals with this by making me throw up‐ there goes the tablet.”	Worries that vomiting, airway/breathing compromise, anxiety/panic, or unconsciousness could make sublingual use unreliable or unsafe, especially given the 1‐min dissolve time. “It's certainly more practical, but what would the effectiveness be in case of vomiting?” “My concern is that a minute is too long. I think my son would spit it out as he's a toddler.” “I'm afraid that during loss of consciousness it would pose a choking risk.”
Preference for choice and layered safety (tablet as an added option)	Rather than replacing existing devices, many framed the tablet as another option; some imagined sequencing/combining (tablet first, injector later) depending on severity and context. “Does not alter my view—I will take adrenaline in any form no matter the route to save my own life” “I would take the tablet first, before injecting myself” “Could I use the injector and then take a pill?” “I'd have the three (nasal, sublingual and injectable adrenaline) at hand in an emergency”	Many wanted additional options but not replacement—keeping injectors available alongside tablet/spray. “That would be amazing and we would use that in addition to having the autoinjector and nasal spray options.” “I would still give my child a pen as well!” “I think the more tools available, the better.”
Acceptability constraints (palatability, child's age, and appropriate‐use concerns)	A smaller cluster raised issues about taste/sensory aversion, excipients/ingredients as potential allergens, tablets getting lost more easily than autoinjectors and potential tablet misuse by non‐allergic individuals—factors that could reduce acceptability for some. “the tablet would have to have a tolerable taste.” “I have a strong gag reflex and sensory aversion to many tastes… I would not want this at all.” “I would worry I would be allergic to the tablet.” “I see a risk of misuse by non‐allergic people.”	Suitability viewed as age‐dependent; cooperation and taste could determine adherence; many felt it was unrealistic for toddlers. Some felt tablets might be easier to get lost or damaged, or more difficult to find by others in an emergency, compared to autoinjector or nasal spray. “My daughter's too little for that… What if she spat it out? Or choked on it?” “I would worry about anything that requires the child to cooperate in an emergency situation when she is distressed.” “Think a child would be more willing… depending on how it tastes.” “If the taste is strong, the child might spit it out or swallow it instead of letting it dissolve.” “The size of the tablet might mean it might get lost of damaged.” “It would be harder for others to find in an emergency.”

## 4 DISCUSSION

To our knowledge, this is the first independent international survey of views on non‐injectable adrenaline for self‐administration. Patients and caregivers prioritized evidence of effectiveness to treat anaphylaxis above other information needs. Notably, respondents were highly concerned about performance in “real‐world” conditions. This included whether nasal symptoms or treatments might affect delivery, highlighting that end‐users seek reassurance not only that ANS works but that it works when reactions are severe, and circumstances are suboptimal. Available adult pharmacokinetic studies provide some reassurance regarding nasal symptoms: allergen‐induced nasal congestion did not impair and may enhance absorption,^13^ while active upper respiratory tract infection had minimal impact on pharmacokinetic or pharmacodynamic responses.^13^, ^14^ However, these findings do not establish clinical effectiveness during severe community reactions. Cost ranked low overall, likely reflecting that many respondents live in countries where patient medication costs are subsidized—in some cases resulting in no out‐of‐pocket expenses for patients. Regulatory approval was rated lower than efficacy/safety for a substantial minority, possibly suggesting a demand for evidence beyond that supporting initial authorisation, lack of familiarity with the regulatory approval process, or limited reassurance from approvals alone. Caregivers of children with food allergy expressed greater concern than food‐allergic adults about correct use of ANS, plausibly reflecting practical realities of treating a child who may resist medication administration and the reliance on non‐parent carers to recognize and respond to anaphylaxis. This aligns with qualitative work showing that food allergy management is highly relational and that parents experience substantial anxiety, hesitation, and decision burden around adrenaline use during reactions.^6^ Notably, free‐text responses suggested that confidence in ANS depends not only on demonstrable efficacy but also on whether the device is recognizable, trusted and usable by bystanders and shared‐care providers such as schools, highlighting an important social‐implementation dimension of emergency treatment choice. This is consistent with current school‐based food allergy/anaphylaxis recommendations and the emphasis on structured training for lay responders.^5^, ^18^ Reassuringly, in simulated emergencies of severe hypoglycaemia for type I diabetes, intranasal glucagon was easier and faster to administer successfully than injectable glucagon for parents/caregivers, school workers and other non‐medically trained users.^19^ This is reflected in current diabetes guidance, which describes intranasal glucagon as part of the newer ready‐to‐use glucagon formulations for community rescue treatment.^20^, ^21^

Alongside these concerns, over half of respondents anticipated using ANS earlier in a reaction and most anticipated always carrying it, consistent with the hypothesis that a needle‐free option may reduce hesitancy and improve readiness to treat. Similar preferences for intranasal over intramuscular rescue treatment have been described in other acute emergencies, such as opioid overdose in adults, where end‐users and lay responders often favor intranasal devices because they are perceived as easier, faster, safer, and more comfortable or less intimidating to administer.^22^ More directly, an industry‐funded, scenario‐based study of 200 adults and 100 caregivers found lower expected anxiety with nasal adrenaline than with an AAI; adults also anticipated better well‐being during a reaction, with larger expected benefits among those with needle phobia.^23^ As in our survey, these findings concern anticipated rather than observed responses and behavior. This is relevant given known gaps in AAI carriage and suboptimal use in the community with barriers extending beyond availability to include recognition, uncertainty, fear, embarrassment, non‐carriage, and practical difficulties in using AAIs during emergencies.^24^, ^25^, ^26^ Consistent with this, embarrassment about AAI use was the only concern that differed by child age, being greater among caregivers of adolescents than among those of younger children. This may reflect increasing sensitivity to peer perceptions during adolescence, although this caregiver‐reported finding requires confirmation.

Poorer food‐allergy‐related HRQoL was associated with greater concern about the effectiveness of both established AAI and emerging ANS. The numerically strongest association in both respondent groups concerned AAI effectiveness, suggesting that treatment‐related worry may reflect a broader burden of food allergy and uncertainty about managing anaphylaxis rather than an information deficit specific to ANS. This broader interpretation is consistent with Polloni et al., whose exploratory factor analysis identified emotional, emergency‐management, and AAI/device‐related dimensions of parental self‐efficacy.^27^ Interventions may therefore need to address wider drivers of HRQoL impairment and treatment‐related worry alongside device‐specific education.

Consistent with the ANS findings, perspectives on sublingual adrenaline showed high acceptability driven by portability and reduced needle‐related barriers, but uptake remained conditional on evidence of comparable speed/effectiveness and on feasibility during reactions (e.g., vomiting, airway compromise, panic, or unconsciousness).

The most popular prescribing preference was to carry both ANS and AAI, supporting a common “hedging bets” approach in low‐information settings,^28^ but also suggesting perceived role differentiation (ANS for earlier use with AAI retained as backup for more severe reactions or when ANS fails). This sits in tension with health‐economic modeling indicating that non‐injectable adrenaline is only cost‐effective versus intramuscular adrenaline if priced within ~US$4 annually, and that routinely adding a non‐injectable device to standard AAI is not cost‐effective due to redundant device costs, even under optimistic assumptions.^29^ This may also reflect how, in time‐critical rescue situations, adoption of a newer route is shaped not by pharmacology alone but by individual trade‐offs between familiarity, perceived ease, speed, safety, and the anticipated emergency context, particularly when lay responders may be involved. Importantly, more “choice” will not necessarily reduce anxiety unless evidence and counseling keep pace: previous work suggests that AAIs can be both reassuring and burdensome; while some studies report reduced anxiety after prescription, others have found lower HRQoL among those prescribed an AAI, possibly reflecting increased treatment burden and perceived risk.^30^, ^31^ These findings reinforce the need for evidence and clear counseling to support shared decision‐making as new delivery options enter clinical practice.

## 5 STRENGTHS AND LIMITATIONS

This survey was conducted at the EU launch of EURneffy, providing early insight into initial impressions and information needs before widespread marketing or anecdotal experience could shape perceptions. Including both adults and caregivers enabled comparison of age‐ and context‐specific priorities relevant to shared decision‐making. However, the open survey design limits verification of respondent identity and allergy status, and dissemination may have favored a predominantly White/Caucasian, highly educated sample with high perceived severity; self‐selection by more anxious or motivated individuals is possible.^32^, ^33^ These characteristics may limit our findings' generalisability to more ethnically and socioeconomically diverse populations. Future studies should use purposive, culturally adapted multilingual recruitment through routine public healthcare and primary‐care services and trusted community organizations beyond established allergy networks, with supported non‐digital participation where needed to reduce language, literacy and digital‐access barriers. Nonetheless, over 90% of participants reported immediate reactions within 2 h and high rates (70%–87%) of prior airway/breathing compromise; all had AAI prescribed, reflecting individuals living with anaphylaxis risk. Since this survey, an industry‐led healthcare professional survey on EURNeffy experience in the USA^34^ and a case series describing allergen‐immunotherapy reactions treated with neffy^35^ have been published, and use in the UK/EU is increasing, though cost remains a barrier; repeating the survey as familiarity grows may yield different patterns.

Finally, a routing error prevented some eligible respondents from accessing the ANS module, substantially reducing its overall completion rate. The marked increase following correction suggests that restricted technical access was the principal driver of the low completion proportion. Module completion also varied by country, likely reflecting differences in the timing and intensity of country‐specific dissemination.

## 6 CONCLUSIONS

These independent patient and caregiver survey data show high interest in emerging non‐injectable adrenaline devices that may address perceived barriers associated with AAIs and could, if confirmed in real‐world studies, improve timely adrenaline use.

However, adults and caregivers emphasized the need for robust evidence on real‐world effectiveness during reactions, including under complicating conditions (e.g., nasal inflammation, vomiting, lack of cooperation). Prescribers, manufacturers and regulators should generate and communicate appropriate efficacy and safety data to meet these information needs. This would support shared decision‐making, informed by clinical evidence, so that expanded device choice reassures rather than amplifies uncertainty.

## AUTHOR CONTRIBUTIONS

**M. F. Gonzalez‐Matamala:** Investigation; writing – original draft; methodology; validation; visualization; writing – review and editing; project administration. **N. Patel:** Conceptualization; writing – original draft; methodology; writing – review and editing. **D. Kahn‐Santoro:** Investigation; methodology; writing – original draft; project administration; writing – review and editing. **R. Knibb:** Conceptualization; writing – review and editing; validation. **G. Roberts:** Conceptualization; writing – review and editing; methodology. **P. J. Turner:** Conceptualization; writing – review and editing; methodology. **A. Kazie:** Investigation; writing – review and editing; methodology. **N. Atzert:** Validation; writing – review and editing; methodology. **J. Bartra:** Conceptualization; methodology; writing – review and editing; supervision. **K. Chetty:** Validation; writing – review and editing; methodology. **A. Deschildre:** Methodology; validation; writing – review and editing. **F. A. Dumitru:** Methodology; validation; writing – review and editing. **W. Feleszko:** Methodology; validation; writing – review and editing. **J. Gerdts:** Methodology; conceptualization; writing – review and editing; validation. **M. H. Gowland:** Methodology; conceptualization; writing – review and editing; validation. **T. T. M. Le:** Methodology; validation; writing – review and editing. **H. Pité:** Methodology; validation; writing – review and editing. **C. Ozdemir:** Methodology; validation; writing – review and editing. **K. Papadopoulou:** Methodology; validation; writing – review and editing. **M. Podestà:** Methodology; conceptualization; validation; writing – review and editing. **C. Raviolo:** Methodology; validation; writing – review and editing. **M. Ruano‐Zaragoza:** Methodology; validation; writing – review and editing. **H. O. Skjerven:** Methodology; validation; writing – review and editing. **B. Stoilov:** Methodology; validation; writing – review and editing. **L. D. Valderrama:** Methodology; validation; writing – review and editing. **C. Venter:** Conceptualization; methodology; validation; writing – review and editing. **C. Woehlk:** Methodology; writing – review and editing. **G. H. Yonamine:** Methodology; validation; writing – review and editing. **M. Vazquez‐Ortiz:** Conceptualization; investigation; funding acquisition; writing – original draft; methodology; writing – review and editing; formal analysis; project administration; data curation; software; supervision; resources.

## FUNDING INFORMATION

This Task force report was supported by the European Academy of Allergy and Clinical Immunology (EAACI) under the EAACI Taskforce ‘FAIR (Food Allergen Ingestion Risk): Understanding accidental food exposure risk in children and young people with food allergies in Europe’ Budget code 40407, 2023–2025.

## CONFLICT OF INTEREST STATEMENT

FGM, DKS, AK, NA, KC, FAD, WF, TML, HP, KP, MP, CR, JB, MRZ, HOS, BS, LDV, CV and GHY declare no conflicts of interest in relation to this manuscript. NP reports research funding from NIHR. RK reports research funding from the National Institute for Health Research, Aimmune, National Peanut Board, Novartis and the Food Standards Agency, and honoraria from Nutricia, Viatris, Stallergenes Greer and DBV Technologies. GR reports research funding from NIHR, NIH, EU IMI and Chiesi; research support from ThermoFisher; and consultancy for ALK‐Abelló, Viatris and AstraZeneca, with honoraria paid to his institution. PJT reports grants from the UK Medical Research Council and the National Institute for Health and Care Research/Imperial Biomedical Research Centre, outside the submitted work, and personal fees from UpToDate and Elsevier, outside the submitted work. AD reports speaker/consulting fees from Aimmune Therapeutics, ALK, AstraZeneca, Celltrion, DBV Technologies, GSK, Novartis, Regeneron Pharmaceuticals Inc., Sanofi, Stallergenes Greer and Viatris, outside the submitted work; AD is also a member of the steering committee of the European Respiratory Society Clinical Research Collaboration SPACE, which has been or is funded by Novartis, AstraZeneca, Sanofi and Celltrion. JG reports that Food Allergy Canada receives unrestricted funding from Viatris, Sanofi and the American Peanut Council to support educational initiatives. MHG has carried adrenaline for emergency use in anaphylaxis since 1978 and reports no other conflicts of interest in relation to this manuscript. CO reports being European Academy of Allergy and Clinical Immunology Allied Health & Primary Care Section Chair and Assistant Editor of *Allergy*. CW is dual affiliated and is currently also employed by the Department of Global Research & Drug Discovery, ALK‐Abelló. MVO reports research funds from National Institute for Health and Care Research, Imperial Biomedical Research Centre NIHR and Food Allergy Research and Education outside the submitted work.

## Supporting information


Figure S1.

Appendix S1.

Table S1.

## Data Availability

The data that support the findings of this study are available on request from the corresponding author. The data are not publicly available due to privacy or ethical restrictions.
