# Supplementary material for: International survey of parental and patient perspectives on emerging adrenaline devices for community‐based management of anaphylaxis: An EAACI task force report
Source: Pediatr Allergy Immunol. 2026 Oct 14;37(10):e70507. doi: 10.1111/pai.70507 (PMC13669523; doi:10.1111/pai.70507)
Supplement: Supplementary file 1 [file PAI-37-e70507-s001.pdf]

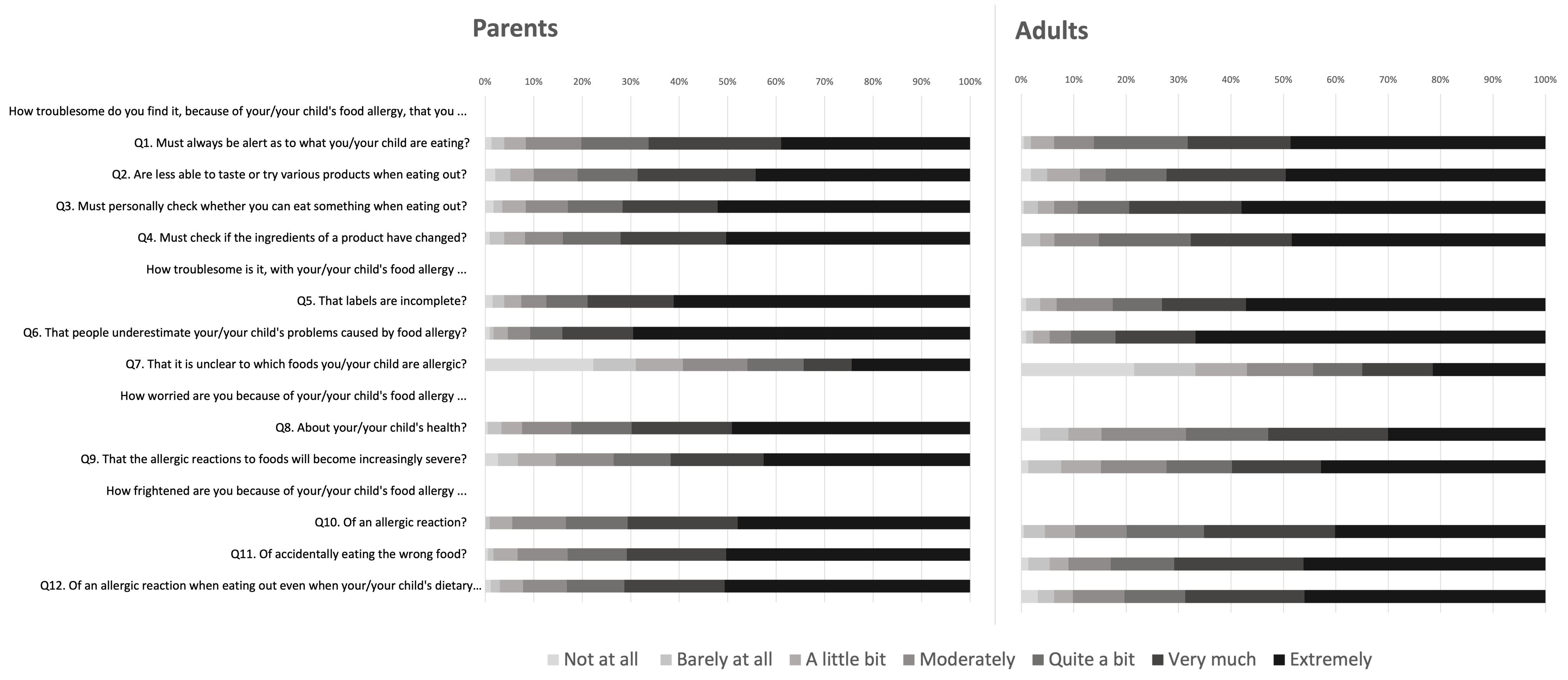

Parents
Adults
0%
10%
20%
30%
40%
50%
60%
70%
80%
90%
100%
How troublesome do you find it, because of your/your child's food allergy, that you ...
Q1. Must always be alert as to what you/your child are eating?
Q2. Are less able to taste or try various products when eating out?
Q3. Must personally check whether you can eat something when eating out?
Q4. Must check if the ingredients of a product have changed?
How troublesome is it, with your/your child's food allergy ...
Q5. That labels are incomplete?
Q6. That people underestimate your/your child's problems caused by food allergy?
Q7. That it is unclear to which foods you/your child are allergic?
How worried are you because of your/your child's food allergy ...
Q8. About your/your child's health?
Q9. That the allergic reactions to foods will become increasingly severe?
How frightened are you because of your/your child's food allergy ...
Q10. Of an allergic reaction?
Q11. Of accidentally eating the wrong food?
Q12. Of an allergic reaction when eating out even when your/your child's dietary...
Not at all
Barely at all
A little bit
Moderately
Quite a bit
Very much
Extremely
