# Supplementary material for: International survey of parental and patient perspectives on emerging adrenaline devices for community‐based management of anaphylaxis: An EAACI task force report
Source: Pediatr Allergy Immunol. 2026 Oct 14;37(10):e70507. doi: 10.1111/pai.70507 (PMC13669523; doi:10.1111/pai.70507)
Supplement: Supplementary file 2 [file PAI-37-e70507-s002.docx]

**Online repository text**

**Methods**

**Thematic analysis**

Reflexive thematic analysis was conducted following Braun & Clarke’s six-phase approach [15] (familiarisation; generating initial codes; searching for themes; reviewing themes; defining/naming themes; write-up). Responses were read and re-read and initial notes were made; data were then coded primarily inductively and at a semantic (explicit) level, to capture participants’ stated concerns, preferences, and information needs. Codes were collated and iteratively clustered into candidate themes, which were repeatedly checked against the coded extracts and the wider dataset to ensure themes were internally coherent, distinct, and captured meaningful patterning across responses. Final themes were defined and named, and reported with concise analytic summaries supported by illustrative verbatim quotes.

**Original surveys**

The original surveys can be found at the bottom of this document

**Results**

**Additional perspectives on worries and information needs regarding ANS and worries about AAI**

Reflexive thematic analysis of the relevant three free-text questions identified recurring patterns across adults and caregivers, with confidence in ANS and AAI shaped by perceived effectiveness, practical feasibility, and the demands of emergency use (Tables E6–E8)

**Worries about ANS**

Responses to the question on worries about ANS (caregivers n = 71, adults n = 27) identified five themes in both adults and caregivers (Table E6): (1) ‘Evidence of equivalence and real-world effectiveness’, capturing concern about whether ANS works as quickly and reliably as AAI, particularly in severe reactions; (2) ‘Route-specific nasal factors and tolerability’, reflecting worries that congestion, blocked nose, epistaxis or local sensitivity could impair delivery or cause adverse effects; (3) ‘Feasibility under reaction conditions’, describing concerns about correct use during panic, breathlessness, tremor, reduced consciousness, or limited child cooperation; (4) ‘Recognition, training, and integration into emergency response’, highlighting uncertainty about whether bystanders, schools and other carers would recognise ANS as an emergency treatment and know when/how to use it; and (5) ‘Implementation and unintended use’, encompassing cost, availability, unfamiliarity, accidental activation, and concern that the lower-threat appearance of a spray might alter thresholds for use. Overall, confidence in ANS was conditional on evidence of effectiveness and practical usability in real emergencies.

**Additional information needs about ANS**

Responses on additional information needs regarding ANS (caregivers n = 108, adults n = 62) identified six themes (Table E7): (1) ‘Evidence of equivalence and real-world effectiveness’, with participants prioritising comparative evidence against intramuscular adrenaline and performance in severe scenarios such as airway compromise or loss of consciousness; (2) ‘Dosing, eligibility, and number of doses’, covering weight- and age-based suitability, adequacy of dose, and whether repeat dosing is possible; (3) ‘Safety profile, ingredients, and contraindications’, including questions about side effects, excipients, allergy risks, and nasal conditions that might affect use; (4) ‘Practicalities’, capturing storage, shelf-life, portability, and device design; (5) ‘Integration into action plans and post-use care’, reflecting uncertainty about when ANS should be used, whether ambulance or hospital attendance remains necessary, and how it fits alongside AAI; and (6) ‘Access, approval, implementation, and recognisability’, describing requests for information on country availability, school use, funding, and risk of confusion with other nasal products. Across both groups, requests were dominated by the need for robust evidence and clear implementation guidance before ANS could be trusted in routine care.

**Worries about AAI**

Responses on worries about AAI (caregivers n = 157, adults n = 77) identified six themes (Table E8): (1) ‘Practical burdens’, describing the ongoing challenges of carriage, storage, expiry and access; (2) ‘Needle fear, pain, and anxiety’ about injection effects, reflecting fear of needles, pain, freezing in the moment, and worries about side effects; (3) ‘Decision-making under uncertainty and post-use pathway burdens’, capturing uncertainty about when a reaction is severe enough to justify use and concerns about ambulance attendance or hospital observation afterwards; (4) ‘Reliability and confidence in others’ ability to administer’, highlighting fears about malfunction and the ability of bystanders, schools or other carers to recognise anaphylaxis and use the device correctly; (5) ‘Managing severe reactions and aftercare logistics’, including concern that one dose may be insufficient or that emergency help may be delayed; and (6) ‘Social restrictions, stigma, and peer pressure’, particularly among caregivers, who described reduced independence, limits on social participation, and concern about future adolescent self-management. Together, these themes showed that worries about AAI extended beyond needle aversion to encompass the wider practical, social and decisional burden of living with injectable emergency treatment.

**Original surveys**

**1. SURVEY ‘Keeping safe with food allergies’ – PARENT OF CHILD WITH FOOD ALLERGY**

**PART 1**

SECTION 1. Background Information:

Information about the respondent

Q1.1. Do you have, or are you the parent or guardian of, a child with food allergy?

a) Yes
b) No
c) Not sure

*(If no/not sure, survey stops)*

Q1.2. What’s your relationship with your child who has food allergy?

a) Mother
b) Father
c) Guardian
d) Other -----------------

Q1.3. What’s your age?

*(number box)*

*(If age <18 years, survey stops)*

Q1.4. What’s the highest level of education you have completed? (List)

a) Primary School
b) Secondary School
c) Vocational or similar
d) University degree
e) Other (please state)

Q1.5. What’s your ethnicity? Choose one option that best describes your ethnic group or background

a) White or Caucasian
b) Black or African American
c) American Indian/ Native American or Alaska Native
d) Asian
e) Mixed
f) Other
g) Prefer not to say

Q1.6. What’s your gender?

a) Male
b) Female
c) Non-binary
d) Prefer not to say

Q1.7. What’s your country of residence? *(dropdown list on Qualtrics)*

- Afghanistan
- Albania
- Algeria
- Andorra
- Angola
- Antigua and Barbuda
- Argentina
- Armenia
- Australia
- Austria
- Azerbaijan
- Bahamas
- Bahrain
- Bangladesh
- Barbados
- Belarus
- Belgium
- Belize
- Benin
- Bhutan
- Bolivia
- Bosnia and Herzegovina
- Botswana
- Brazil
- Brunei Darussalam
- Bulgaria
- Burkina Faso
- Burundi
- Cambodia
- Cameroon
- Canada
- Cape Verde
- Central African Republic
- Chad
- Chile
- China
- Colombia
- Comoros
- Congo, Republic of the...
- Costa Rica
- Côte d'Ivoire
- Croatia
- Cuba
- Cyprus
- Czech Republic
- Democratic Republic of the Congo
- Denmark
- Dibouti
- Dominica
- Dominican Republic
- Ecuador
- Egypt
- El Salvador
- Equatorial Guinea
- Eritrea
- Estonia
- Ethiopia
- Fiji
- Finland
- France
- Gabon
- Gambia
- Georgia
- Germany
- Ghana
- Greece
- Grenada
- Guatemala
- Guinea
- Guinea-Bissau
- Guyana
- Haiti
- Honduras
- Hong Kong (S.A.R.)
- Hungary
- Iceland
- India
- Indonesia
- Iran
- Iraq
- Ireland
- Israel
- Italy
- Jamaica
- Japan
- Jordan
- Kazakhstan
- Kenya
- Kiribati
- Kuwait
- Kyrgyzstan
- Lao People's Democratic Republic
- Latvia
- Lebanon
- Lesotho
- Liberia
- Libyan Arab Jamahiriya
- Liechtenstein
- Lithuania
- Luxembourg
- Madagascar
- Malawi
- Malaysia
- Maldives
- Mali
- Malta
- Marshall Islands
- Mauritania
- Mauritius
- Mexico
- Micronesia, Federated States of...
- Monaco
- Mongolia
- Montenegro
- Morocco
- Mozambique
- Myanmar
- Namibia
- Nauru
- Nepal
- Netherlands
- New Zealand
- Nicaragua
- Niger
- Nigeria
- North Korea
- Norway
- Oman
- Pakistan
- Palau
- Panama
- Papua New Guinea
- Paraguay
- Peru
- Philippines
- Poland
- Portugal
- Qatar
- Republic of Moldova
- Romania
- Russian Federation
- Rwanda
- Saint Kitts and Nevis
- Saint Lucia
- Saint Vincent and the Grenadines
- Samoa
- San Marino
- Sao Tome and Principe
- Saudi Arabia
- Senegal
- Serbia
- Seychelles
- Sierra Leone
- Singapore
- Slovakia
- Slovenia
- Solomon Islands
- Somalia
- South Africa
- South Korea
- Spain
- Sri Lanka
- Sudan
- Suriname
- Swaziland
- Sweden
- Switzerland
- Syrian Arab Republic
- Tajikistan
- Thailand
- The former Yugoslav Republic of Macedonia
- Timor-Leste
- Togo
- Tonga
- Trinidad and Tobago
- Tunisia
- Turkey
- Turkmenistan
- Turkey
- Turkmenistan
- Tuvalu
- Uganda
- Ukraine
- United Arab Emirates
- United Kingdom of Great Britain and Northern Ireland
- United Republic of Tanzania
- United States of America
- Uruguay
- Uzbekistan
- Vanuatu
- Venezuela, Bolivarian Republic of...
- Viet Nam
- Yemen
- Zambia
- Zimbabwe

Information about the child

Q1.8.1. How many children do you have/care for with food allergy?

a) 1
b) 2
c) 3
d) 4
e) 5
f) >5

*(If answer option a selected, then please move on to Q1.8.2., otherwise please continue from Q1.9. onwards)*

Q1.8.2.Are your children you care for allergic to the same foods?

- Yes, they are allergic to the same foods
- No, they are allergic to different foods
- No, some of their food allergies are the same and some are different

*If you care for more than one child with food allergies, we would like you to answer the survey thinking about your child who has had the highest number of allergic reactions in the last 3 years.*

Q1.9. What’s your child’s current age in years?

a) 0
b) 1
c) 2
d) 3
e) 4
f) 5
g) 6
h) 7
i) 8
j) 9
k) 10
l) 11
m) 12
n) 13
o) 14
p) 15
q) 16
r) 17
s) 18
t) >18

Q1.10. At what age (in years) was your child first diagnosed with food allergy?

a) 0
b) 1
c) 2
d) 3
e) 4
f) 5
g) 6
h) 7
i) 8
j) 9
k) 10
l) 11
m) 12
n) 13
o) 14
p) 15
q) 16
r) 17
s) 18
t) >18

Q1.11. Was your child’s food allergy diagnosed by a healthcare professional?

a) Yes
b) No
c) Not sure

Q1.12. Which of the following tests (if any) did your child have for their food allergy? (Tick all that apply)

a) Skin prick test
b) Blood test for IgE
c) Trying the food under supervision of a healthcare professional (e.g. Oral food challenge or supervised feed test)
d) Other tests (please describe – free text-)
e) My child has had no tests for food allergy
f) Not sure

Q1.13. How severe do you think your child’s food allergy is?

a) Mild
b) Moderate
c) Severe
d) Not sure

Q1.14. Has your child had an unxpected reaction to a food within 2 hours of eating?

a) Yes
b) No
c) Not sure

Q1.15. Has your child ever had a reaction(s) to a food involving any of the following? (tick all that apply)

a) Airway problem (e.g. hoarse voice, difficulty swallowing, swollen tongue)
b) Breathing problem (e.g. difficult or noisy breathing, wheeze or persistent cough)
c) Consciousness problem (e.g. Persistent dizziness, pale or floppy, suddenly sleepy, collapse/unconscious)
d) None of the above

Q1.16. Does your child have an allergy emergency action plan?

a) Yes
b) No
c) Not sure

Q1.17.1. Does your child have an adrenaline autoinjector(s) (e.g. Epipen, Jext)?

a) Yes
b) No
c) Not sure

*(If answer option a selected, then please move on to Q1.17.2., otherwise please continue from Q1.18. onwards)*

Q.17.2. Have you been shown how to use the adrenaline autoinjector? (tick all that apply)

a) Yes, a healthcare professional showed me how to use i
b) Yes, I have watched a video online or similar
c) No
d) Not sure

Q1.18. What food(s) is your child allergic to? (Tick all that apply)

a) Cow’s milk/Dairy
b) Hen’s Egg
c) Peanut
d) Tree nuts (e.g. hazelnut, almond, walnut, Brazil nut, cashew nut, pistachio nut, etc)
e) Sesame
f) Mustard
g) Other seeds (e.g. sunflower seeds, pumpkin seds, linseeds, poppy seed, pine nuts, etc)
h) Fish
i) Shellfish (e.g. prawns, lobster, mussels, squid, clams, etc)
j) Soya
k) Lupin
l) Other legumes or pulses (e.g. pea, lentil, beans, etc)
m) Wheat/Grains containing gluten (e.g. rye, barley, wheat, etc)
n) Other grains (e.g. rice, corn, oat, etc)
o) Vegetables
p) Fruit (e.g. apple, peach, banana, etc)
q) Meat/chicken
r) Food additives
s) Other (free text)

**SECTION 2. ALLERGIC REACTIONS (ONLY QUESTIONS COVERED IN THIS MANUSCRIPT ARE SHOWN)**

Q2.1.Has your child had any allergic reactions to foods by accident in the last 3 years ?

a) Yes
b) No
c) Not Sure

*(If no, skip all remaining questions in section 2 and move on to section 3)*

Q2.2.Please state the number of reactions uour child has had in the last 3 years:

a) 1
b) 2
c) 3
d) 4
e) 5
f) >5

Q2.3.Has your child needed injectable adrenaline (e.g. Epipen or Jext) for any of those reactions?

a) Yes
b) No
c) Not sure

SECTION 3 (OMITTED AS NOT COVERED IN THIS MANUSCRIPT)

SECTION 4. Preparedness and challenges when trying to make safe food choices (ONLY QUESTION(S) COVERED IN THIS MANUSCRIPT ARE SHOWN)

Q4.1.Overall, how confident do you feel about managing food allergy in your daily life?

*(scale)*

a) Not at all confident
b) A little confident
c) Somewhat confident
d) Very confident
e) Extremely confident
f) Don’t know

SECTION 5. QUALITY OF LIFE

The following are scenarios that parents have told us affect children’s quality of life because of food allergy. Please indicate how much of an impact each scenario has on **your child’s** quality of life by ticking in one of the boxes numbered 0-6.

| **0** | **1** | **2** | **3** | **4** | **5** | **6** |
| --- | --- | --- | --- | --- | --- | --- |
| not at all | barely at all | a little bit | moderately | quite a bit | very much | extremely |

How troublesome do you find it, because of your child’s food allergy, that you ...

Q5.1. Must always be alert as to what they are eating?

Q5.2. Are less able to taste or try various products when eating out?

Q5.3. Must personally check whether your child can eat something when eating out?

Q5.4. That the ingredients of a product change?

How troublesome is it, because of your child’s food allergy ...

Q5.5. That labels are incomplete?

Q5.6. That people underestimate your child’s problems caused by food allergy?

Q5.7. That it is unclear to which foods your child is allergic?

How worried are you because of your child’s food allergy ...

Q5.8. About their health?

Q5.9. That their allergic reactions to foods will become increasingly severe?

How frightened are you because of your child’s food allergy ...

Q5.10.Of an allergic reaction?

Q5.11.Of accidentally eating the wrong food?

Q5.12.Of an allergic reaction when eating out even when your child’s dietary restrictions have been discussed beforehand?

**PART 2** (OMITTED AS NOT COVERED IN THIS MANUSCRIPT)

**PART 3.** USE OF ADRENALINE

SECTION 1: ADRENALINE AUTOINJECTORS

Q1. Has your child ever been prescribed an adrenaline autoinjector by a healthcare professional?

a) Yes
b) No
c) Not sure
d) Other – Please specify (free text)

*(If reply is ‘No’, survey stops here - Thank you for taking the time to complete the survey’)*

*(If reply is ‘Yes’, the following information and questions will come up)*

This next section asks about your experiences and views with regards to using adrenaline to treat allergic reactions in your child. There are some questions about adrenaline autoinjectors- these are also known as adrenaline injectable ‘pens’, examples are Epipen, Jext, Auvi-Q, Anapen. Following these, there are some questions about a new adrenaline nose spray which has been approved in USA and Europe (but not yet the UK) as an alternative to an adrenaline injection.

Q2. Has your child ever been treated with an adrenaline autoinjector for an allergic reaction? (tick all that apply)

a) Yes, I have used the adrenaline autoinjector on my child
b) Yes, someone has administered the adrenaline autoinjector to my child
c) No
e) Other – Please specify (free text)

Q3. How often do you/your child have their adrenaline autoinjector with them (i.e. so they could use immediately if they experience anaphylaxis)? (tick one)

a) Always
b) Most of the time
c) About half the time
d) Occasionally
e) Never
f) Don’t know

Q4. We would like to hear your views on the following issues in a situation where your child needed to use an adrenaline autoinjector to treat a reaction in the future. How would you feel about…

(Scale)

X:

a) Not at all worried
b) A little worried
c) Somewhat worried
d) Very worried
e) Extremely worried
f) Note sure

Y:

Q4.1. knowing how to correctly use the autoinjector?

Q4.2. knowing when to use the autoinjector?

Q4.3. whether other people who come to help you will know when and how to correctly use the autoinjector?

Q4.4. the autoinjector possibly being painful for your child?

Q4.5. the autoinjector causing other side effects?

Q4.6. the autoinjector not working well enough to stop your child’s reaction?

Q4.7. You being too embarrassed to use the autoinjector?

Q4.8. Your child being too embarrassed to use the autoinjector?

Q4.9. You being too scared to use the autoinjector?

Q4.10. Your child being too scared to use the autoinjector?

Q4.11. the autoinjector not being easy or practical to carry around for you or your child?

Q5. Tell us about any other worries you have about using (or your child using) an adrenaline autoinjector (Free text)

**SECTION 2: ADRENALINE NOSE SPRAY**

A new nose spray containing adrenaline has been developed. This allows people to self-treat a serious allergic reaction (anaphylaxis) by spraying adrenaline into their nose rather than injecting adrenaline into their leg muscle. The American (FDA) and European (EMA) medicine regulatory authorities have approved this new treatment recently (meaning doctors will be able to prescribe the medicine to allergic people soon). This is based on data that shows that squirting adrenaline into the nose gives similar adrenaline levels in the blood as an adrenaline autoinjector. The nasal spray is 2.25 in x 1.75 in x 0.75 in (5.7cm x 4.4cm x 1.9cm) in size.

Q1. How important would it be for you to know about each of the following aspects to help you decide if you prefer for your child to have the adrenaline nose spray medication rather than an adrenaline autoinjector in their emergency medicine bag?

(Scale)

X:

a) Not important at all.
b) A little important
c) Moderately important
d) Very important
e) Extremely important
f) Not sure
g) I don’t want to consider an adrenaline nasal spray

Y:

Q1.1. How the nasal spray works?

Q1.2. Whether the nasal spray has had approval in your country?

Q1.3. How effective the nasal spray has been in treating people experiencing severe allergic reactions (anaphylaxis)?

Q1.4. How effective the nose spray is compared to an injectable autoinjector?

Q1.5. Whether the side effects for the nasal spray are more common or more severe than those for the autoinjector?

Q1.6. Whether how well the spray works is affected by child having a problem with their nose (e.g. they have bad hay fever or are also using other nose spray medicines)?

Q1.7. Time to expiry date of the nose spray compared to the autoinjector?

Q1.8. How much the nose spray will cost you compared to an autoinjector?

Q1.9. How portable or easily storable the nose spray is compared to the autoinjector?

Q1.10. How environmentally friendly the nose spray is compared to the autoinjector?

Q2. Can you tell us about any additional information you would like to have about the adrenaline nasal spray? (Free text)

Q3. We would like to hear your views on using an adrenaline nose spray for an allergic reaction (in your child) in the future. Please rate how you would feel about the following aspects:

(Scale)

X:

a) Not at all worried
b) A little worried
c) Somewhat worried
d) Very worried
e) Extremely worried
f) Not sure

Y:

Q3.1. knowing how to use the nasal spray?

Q3.2. knowing when to use the nasal spray?

Q3.3. whether other people who come to help your child will know when and how to correctly use the nasal spray?

Q3.4. the spray possibly being painful for your child?

Q3.5. the spray causing other side effects?

Q3.6. the spray not working well enough to stop your child’s reaction?

Q3.7. the spray not working as well as the adrenaline autoinjector to stop your child’s reaction?

Q3.8. You being too embarrassed to use the nasal spray?

Q3.9. Your child being too embarrassed to use the nasal spray?

Q3.10. You being too scared to use the nasal spray?

Q3.11. Your child being too scared to use the nasal spray?

Q3.12. the spray not being easy or practical to carry around for you or your child?

Q4. Please tell us about any other worries you have about using (or your child using) an adrenaline nose spray to treat an allergic reaction? (Free text)

Q5. If you were prescribed an adrenaline nose spray, how often do you think you/your child would have it with your child (i.e. so they could use immediately if they experience anaphylaxis)?

a) Always
b) Most of the time
c) About half the time
d) Occasionally
e) Never
f) Don’t know

Q6. Please rate how comfortable you would feel about using an adrenaline nasal spray in the future compared to using an adrenaline autoinjector to treat an allergic reaction in your child? I’d feel…

a) Much less comfortable
b) A little less comfortable
c) As comfortable
d) A little more comfortable
e) Much more comfortable
f) Not sure

Q7. Please rate how comfortable you think your child would feel about using an adrenaline nasal spray in the future compared to using an adrenaline autoinjector to treat an allergic reaction in themselves? My child would feel… (tick one)

a) Much less comfortable
b) A little less comfortable
c) As comfortable
d) A little more comfortable
e) Much more comfortable
f) Not sure

Q8. Do you think that the type of treatment (nasal spray or autoinjector) your child had would affect how readily you would use adrenaline during an anaphylaxis reaction? (tick one)

a) Yes, I would use the nose spray earlier than the autoinjector
b) Yes, I would wait longer to use the nose spray compared to the autoinjector
c) No, whichever I had would not affect when I decided to treat myself
d) No, I would wait for medical help to arrive and not use autoinjector or spray before then
e) Not sure
f) Other – please specify (free text)

Q9. Do you think that the type of treatment (nasal spray or autoinjector) your child had would affect how readily they would use adrenaline during an anaphylaxis reaction? (tick one)

a) My child is too young to manage an allergic reaction and use either
b) Yes, they would use the nose spray earlier than the autoinjector
c) Yes, they would wait longer to use the nose spray compared to the autoinjector
d) No, whichever they had would not affect when they decided to treat myself
e) No, I would wait for medical help to arrive and not use autoinjector or spray before then
f) Not sure
g) Other – please specify (free text)

Q10. With the information you have at the moment, if you were offered a prescription for an adrenaline nasal spray for your child by their healthcare team, what would you prefer? (Tick one)

a) I’d prefer my child had an adrenaline nasal spray(s) only
b) I’d prefer my child had an adrenaline autoinjector(s) only
c) I’d prefer my child had an adrenaline autoinjector(s) only but I’d consider nasal spray when more data/information becomes available
d) I’d prefer for my child to have both adrenaline autoinjector(s) and nasal spray(s) with them
e) Not sure
f) Other (free text)

13. Please tell us how any of your views might change if, instead of a nose spray, the new treatment available was an adrenaline tablet which your child placed under their tongue for about 1 minute. The tablet would dissolve in their mouth and deliver adrenaline to their body via their blood stream without the need for an injection. (Free text)

Thank you for taking the time to complete the survey

**SURVEY ‘Keeping safe with food allergies’ – PATIENT AGED 16 AND OVER**

**PART 1**

SECTION 1. Background Information

Information about the respondent

Q1.1. Do you have food allergy?

a) Yes
b) No
c) Not sure

*(If no/not sure, survey stops)*

Q1.2. What’s your age?

(*number box)*

*(If age <16 years, survey stops)*

Q1.3. What’s the highest level of education you have completed?

a) Primary School
b) Secondary School
c) Vocational or similar
d) University degree
e) Other (please state)

Q1.4. What’s your ethnicity? Choose one option that best describes your ethnic group or background

a) White or Caucasian
b) Black or African American
c) American Indian/ Native American or Alaska Native
d) Asian
e) Mixed
f) Other
g) Prefer not to say

Q1.5. What’s your gender? Male, female, non-binary, Prefer not to say

Q1.6. What’s your country of residence? *(dropdown list on Qualtrics)*

- Afghanistan
- Albania
- Algeria
- Andorra
- Angola
- Antigua and Barbuda
- Argentina
- Armenia
- Australia
- Austria
- Azerbaijan
- Bahamas
- Bahrain
- Bangladesh
- Barbados
- Belarus
- Belgium
- Belize
- Benin
- Bhutan
- Bolivia
- Bosnia and Herzegovina
- Botswana
- Brazil
- Brunei Darussalam
- Bulgaria
- Burkina Faso
- Burundi
- Cambodia
- Cameroon
- Canada
- Cape Verde
- Central African Republic
- Chad
- Chile
- China
- Colombia
- Comoros
- Congo, Republic of the...
- Costa Rica
- Côte d'Ivoire
- Croatia
- Cuba
- Cyprus
- Czech Republic
- Democratic Republic of the Congo
- Denmark
- Dibouti
- Dominica
- Dominican Republic
- Ecuador
- Egypt
- El Salvador
- Equatorial Guinea
- Eritrea
- Estonia
- Ethiopia
- Fiji
- Finland
- France
- Gabon
- Gambia
- Georgia
- Germany
- Ghana
- Greece
- Grenada
- Guatemala
- Guinea
- Guinea-Bissau
- Guyana
- Haiti
- Honduras
- Hong Kong (S.A.R.)
- Hungary
- Iceland
- India
- Indonesia
- Iran
- Iraq
- Ireland
- Israel
- Italy
- Jamaica
- Japan
- Jordan
- Kazakhstan
- Kenya
- Kiribati
- Kuwait
- Kyrgyzstan
- Lao People's Democratic Republic
- Latvia
- Lebanon
- Lesotho
- Liberia
- Libyan Arab Jamahiriya
- Liechtenstein
- Lithuania
- Luxembourg
- Madagascar
- Malawi
- Malaysia
- Maldives
- Mali
- Malta
- Marshall Islands
- Mauritania
- Mauritius
- Mexico
- Micronesia, Federated States of...
- Monaco
- Mongolia
- Montenegro
- Morocco
- Mozambique
- Myanmar
- Namibia
- Nauru
- Nepal
- Netherlands
- New Zealand
- Nicaragua
- Niger
- Nigeria
- North Korea
- Norway
- Oman
- Pakistan
- Palau
- Panama
- Papua New Guinea
- Paraguay
- Peru
- Philippines
- Poland
- Portugal
- Qatar
- Republic of Moldova
- Romania
- Russian Federation
- Rwanda
- Saint Kitts and Nevis
- Saint Lucia
- Saint Vincent and the Grenadines
- Samoa
- San Marino
- Sao Tome and Principe
- Saudi Arabia
- Senegal
- Serbia
- Seychelles
- Sierra Leone
- Singapore
- Slovakia
- Slovenia
- Solomon Islands
- Somalia
- South Africa
- South Korea
- Spain
- Sri Lanka
- Sudan
- Suriname
- Swaziland
- Sweden
- Switzerland
- Syrian Arab Republic
- Tajikistan
- Thailand
- The former Yugoslav Republic of Macedonia
- Timor-Leste
- Togo
- Tonga
- Trinidad and Tobago
- Tunisia
- Turkey
- Turkmenistan
- Turkey
- Turkmenistan
- Tuvalu
- Uganda
- Ukraine
- United Arab Emirates
- United Kingdom of Great Britain and Northern Ireland
- United Republic of Tanzania
- United States of America
- Uruguay
- Uzbekistan
- Vanuatu
- Venezuela, Bolivarian Republic of...
- Viet Nam
- Yemen
- Zambia
- Zimbabwe

Q1.7. At what age (in years) were you first diagnosed with food allergy?

a) 0
b) 1
c) 2
d) 3
e) 4
f) 5
g) 6
h) 7
i) 8
j) 9
k) 10
l) 11
m) 12
n) 13
o) 14
p) 15
q) 16
r) 17
s) 18
t) >18

Q1.8. Was your food allergy diagnosed by a healthcare professional?

a) Yes
b) No
c) Not sure

Q1.9. Which of the following tests (if any) did you have for your food allergy? (Tick all that apply)

a) Skin prick test
b) Blood test for IgE
c) Trying the food under supervision of a healthcare professional (e.g. Oral food challenge or supervised feed test)
d) Other tests (please describe – free text-)
e) Not sure

Q1.10. How severe do you think your food allergy is?

a) Mil
b) Moderate
c) Severe
d) Not sure

Q1.11. Have you had an unexpected reaction to a food within 2 hours of eating?

a) Yes
b) No
c) Not sure

Q1.12. Have you ever had a reaction(s) to a food involving any of the following? (tick all that apply)

a) Airway problem (e.g. hoarse voice, difficulty swallowing, swollen tongue)
b) Breathing problem (e.g. difficult or noisy breathing, wheeze or persistent cough)
c) Consciousness problem (e.g. Persistent dizziness, pale or floppy, suddenly sleepy, collapse/unconscious)
d) None of the above

Q1.13. Do you have an allergy emergency action plan?

a) Yes
b) No
c) Not sure

Q1.14.1. Do you have an adrenaline autoinjector(s) (e.g. Epipen, Jext)?

a) Yes
b) No
c) Not sure

*(if no or not sure then skip Q.1.14.2. and move onto Q.1.15.)*

Q.1.14.2. Have you been shown how to use the adrenaline autoinjector? (tick all that apply)

a) Yes – a healthcare professional showed me how to use it OR I have watched a video online OR similar
b) No
c) Not sure

Q1.15. What food(s) are you allergic to? (Tick all that apply)

a) Cow’s milk/Dairy
b) Hen’s Egg
c) Peanut
d) Tree nuts (e.g. hazelnut, almond, walnut, Brazil nut, cashew nut, pistachio nut, etc)
e) Sesame
f) Mustard
g) Other seeds (e.g. sunflower seeds, pumpkin seds, linseeds, poppy seed, pine nuts, etc)
h) Fish
i) Shellfish (e.g. prawns, lobster, mussels, squid, clams, etc)
j) Soya
k) Lupin
l) Other legumes or pulses (e.g. pea, lentil, beans, etc)
m) Wheat/Grains containing gluten (e.g. rye, barley, wheat, etc)
n) Other grains (e.g. rice, corn, oat, etc)
o) Vegetables
p) Fruit (e.g. apple, peach, banana, etc)
q) Meat/chicken
r) Food additives
s) Other (free text)

SECTION 2. ALLERGIC REACTIONS (ONLY QUESTIONS COVERED IN THIS MANUSCRIPT ARE SHOWN)

Q2.1.Have you had any allergic reactions to foods by accident in the last 3 years ?

a) Yes
b) No
c) Not Sure

*(If no, skip all remaining questions in section 2 and move on to section 3)*

Q2.2.Please state the number of reactions you have had in the last 3 years:

a) 1
b) 2
c) 3
d) 4
e) 5
f) >5

Q2.3.Have you needed injectable adrenaline (e.g. Epipen or Jext) for any of those reactions?

a) Yes
b) No
c) Not sure

SECTION 3 (OMITTED AS NOT COVERED IN THIS MANUSCRIPT)

SECTION 4. Preparedness and challenges when trying to make safe food choices (ONLY QUESTION(S) COVERED IN THIS MANUSCRIPT ARE SHOWN)

Q4.1.Overall, how confident do you feel about managing food allergy in your daily life?

*(Scale)*

a) Not at all confident
b) A little confident
c) Somewhat confident
d) Very confident
e) Extremely confident
f) Don’t know

SECTION 5. QUALITY OF LIFE

The following are scenarios that adults have told us affect their quality of life because of food allergy. Please indicate how much of an impact each scenario has on your quality of life by ticking in one of the boxes numbered 0-6.

| **0** | **1** | **2** | **3** | **4** | **5** | **6** |
| --- | --- | --- | --- | --- | --- | --- |
| not at all | barely at all | a little bit | moderately | quite a bit | very much | extremely |

How troublesome do you find it, because of your food allergy, that you ...

Q5.1. Must always be alert as to what you are eating?

Q5.2. Are less able to taste or try various products when eating out?

Q5.3. Must personally check whether you can eat something when eating out?

Q5.4. That the ingredients of a product change?

How troublesome is it, because of your food allergy ...

Q5.5. That labels are incomplete?

Q5.6. That people underestimate your problems caused by food allergy?

Q5.7. That it is unclear to which foods you are allergic?

How worried are you because of your food allergy ...

Q5.8. About your health?

Q5.9. That the allergic reactions to foods will become increasingly severe?

How frightened are you because of your food allergy ...

Q5.10.Of an allergic reaction?

Q5.11.Of accidentally eating the wrong food?

Q5.12.Of an allergic reaction when eating out even when your dietary restrictions have been discussed beforehand?

**PART 2** (OMITTED AS NOT COVERED IN THIS MANUSCRIPT)

**PART 3.** USE OF ADRENALINE

SECTION 1: ADRENALINE AUTOINJECTORS

Q1. Have you been prescribed an adrenaline autoinjector by a healthcare professional?

f) Yes
g) No
h) Not sure
i) Other – Please specify: free text

*(If reply is ‘No’, survey stops here - Thank you for taking the time to complete the survey’)*

*(If reply is ‘Yes’, the following information and questions will come up)*

This next section asks about your experiences and views with regards to using adrenaline to treat allergic reactions. There are some questions about adrenaline autoinjectors- these are also known as adrenaline injectable ‘pens’, examples are Epipen, Jext, Auvi-Q, Anapen. Following these, there are some questions about a new adrenaline nose spray which has been approved in USA and Europe (but not yet the UK) as an alternative to an adrenaline injection.

Q.2. Have you ever been treated with an adrenaline autoinjector for an allergic reaction? (tick all that apply)

a) Yes, I have used the adrenaline autoinjector on myself
b) Yes, someone has administered the adrenaline autoinjector to me
c) No
j) Other – Please specify: free text

Q3. How often do you have your adrenaline autoinjector pens with you (i.e. so you could use immediately if you experience anaphylaxis)?

a) Always
b) Most of the time
c) About half the time
d) Occasionally
e) Never
f) Don’t know

Q4. We would like to hear your views on how you would feel about the following issues in a situation where you need to use an adrenaline autoinjector to treat a reaction in the future. How would you feel about…

(Scale)

X:

a) Not at all worried
b) A little worried
c) Somewhat worried
d) Very worried
e) Extremely worried
f) Note sure

Y:

Q4.1. knowing how to correctly use the autoinjector?

Q4.2. knowing when to use the autoinjector?

Q4.3. whether other people who come to help me will know when and how to correctly use the autoinjector?

Q4.3. the autoinjector possibly being painful when used?

Q4.4. the autoinjector causing other side effects?

Q4.5. the autoinjector not working well enough to stop my reaction?

Q4.6. being too embarrassed to use the autoinjector?

Q4.7. being too scared to use the autoinjector?

Q4.8. the autoinjector not being easy or practical to carry around?

Q5.Tell us about any other worries you have about using an adrenaline autoinjector (Free text)

SECTION 2: ADRENALINE NOSE SPRAY

A new nose spray containing adrenaline has been developed. This allows people to self-treat a serious allergic reaction (anaphylaxis) by spraying adrenaline into their nose rather than injecting adrenaline into their leg muscle. The American (FDA) and European (EMA) medicine regulatory authorities have approved this new treatment recently (meaning doctors will be able to prescribe the medicine to people with allergies soon). This is based on data that shows that squirting adrenaline into the nose gives similar adrenaline levels in the blood as an adrenaline autoinjector. The nasal spray is 2.25 in x 1.75 in x 0.75 in (5.7cm x 4.4cm x 1.9cm) in size.

Q1. How important would it be for you to know about each of the following aspects to help you decide if you prefer to have the adrenaline nose spray medication rather than an adrenaline autoinjector in your emergency medicine bag?

(Scale)

X:

h) Not important at all.
i) A little important
j) Moderately important
k) Very important
l) Extremely important
m) Not sure
n) I don’t want to consider an adrenaline nasal spray

Y

Q1.1. How the nasal spray works?

Q1.2. Whether the nasal spray has had approval in your country?

Q1.2. How effective the nasal spray has been in treating people experiencing severe allergic reactions (anaphylaxis)?

Q1.3. How effective the nose spray is compared to an autoinjector?

Q1.4. Whether the side effects for the nasal spray are more common or more severe than those for the autoinjector?

Q1.5. Whether how well the spray works is affected by having a problem with your nose (e.g. if hay fever is bad or I’m are using other nose spray medicines)?

Q1.6. Time to expiry date of the nose spray compared to the autoinjector?

Q1.7. How much the nose spray will cost you compared to an autoinjector?

Q1.8. How portable or easily storable the nose spray is compared to the autoinjector?

Q1.9. How environmentally friendly the nose spray is compared to the autoinjector?

Q2. Can you tell us about any additional information you would like to have about the adrenaline nasal spray? Free text

Q3. We would like to hear your views on using an adrenaline nose spray for an allergic reaction in the future. Please rate how you would feel about the following aspects:

(Scale)

X:

a) Not at all worried
b) A little worried
c) Somewhat worried
d) Very worried
e) Extremely worried
f) Not sure

Y:

Q3.1. knowing how to use the nasal spray?

Q3.2. knowing when to use the nasal spray?

Q3.3. whether other people who come to help me will know when and how to correctly use the nasal spray?

Q3.4. the spray being painful?

Q3.5. the spray causing other side effects?

Q3.6. the spray not working well enough to stop my reaction?

Q3.7. the spray not working as well as the adrenaline autoinjector to stop my reaction?

Q3.8. being too embarrassed to use the nasal spray?

Q3.9. being too scared to use the nasal spray?

Q3.10. the nasal spray not being easy or practical to carry around?

Q4. Please tell us about any other worries you may have about using an adrenaline nose spray to treat an allergic reaction (Free text)

Q5. If you were prescribed an adrenaline nose spray, how often do you think you would have it with you (i.e. so you could use immediately if you experience anaphylaxis)?

a) Always
b) Most of the time
c) About half the time
d) Occasionally
e) Never
f) Don’t know

Q6. Please rate how comfortable you would feel about using an adrenaline nasal spray in the future compared to using an adrenaline autoinjector to treat an allergic reaction? I’d feel…

a) Much less comfortable
b) A little less comfortable
c) As comfortable
d) A little more comfortable
e) Much more comfortable
f) Not sure

Q7. Do you think that the type of treatment(nasal spray or autoinjector) you have would affect how readily you would use adrenaline during an anaphylaxis reaction?

a) Yes, I would use the nose spray earlier than the autoinjector
b) Yes, I would wait longer to use the nose spray compared to the autoinjector
c) No, whichever I had would not affect when I decided to treat myself
d) No, I would wait for medical help to arrive and not use autoinjector or spray before then
e) Not sure
f) Other – Please specify (free text)

Q8. With the information you have at the moment, if you were offered a prescription for an adrenaline nasal spray by your healthcare team, what would you prefer?

a) I’d prefer to have adrenaline nasal spray(s) only
b) I’d prefer to have adrenaline autoinjector(s) only
c) I’d prefer to have adrenaline autoinjector(s) only but I’d consider nasal spray when more data/information becomes available
d) I’d prefer to have both adrenaline autoinjector(s) and nasal spray(s) with me
e) Not sure
g) Other – Please specify (free text)

Q9. Please tell us how any of your views might be altered if, instead of a nose spray, the new treatment available was an adrenaline tablet which you placed under your tongue. The tablet would take about 1 minute to dissolve in your mouth and would deliver adrenaline to your body via your blood stream without the need for an injection (Free text)

Thank you for taking the time to complete the survey
