# Supplementary material for: International survey of parental and patient perspectives on emerging adrenaline devices for community‐based management of anaphylaxis: An EAACI task force report
Source: Pediatr Allergy Immunol. 2026 Oct 14;37(10):e70507. doi: 10.1111/pai.70507 (PMC13669523; doi:10.1111/pai.70507)
Supplement: Supplementary file 3 [file PAI-37-e70507-s003.zip › Table E10.docx]

**Table E10. Information needs, AAI- and ANS-related concerns, and anticipated ANS use and preferences according to respondent age: patient survey**

| **Domain** | **Survey item** | **Unadjusted p** | **Holm- adjusted p** |
| --- | --- | --- | --- |
| **AAI concerns** | Knowing how to use the AAI correctly | 0.218 | 1.000 |
|  | Knowing when to use the AAI | 0.759 | 1.000 |
|  | Whether other people would know when and how to use the AAI | 0.900 | 1.000 |
|  | AAI possibly being painful | 0.002 | 0.081 |
|  | AAI causing other side effects | 0.967 | 1.000 |
|  | AAI not working well enough | 0.622 | 1.000 |
|  | Being too embarrassed to use the AAI | 0.232 | 1.000 |
|  | Being too scared to use the AAI | 0.069 | 1.000 |
|  | AAI not being easy or practical to carry | 0.042 | 1.000 |
| **ANS information needs** | How the ANS works | 0.943 | 1.000 |
|  | Whether the ANS has regulatory approval in the respondent's country | 0.517 | 1.000 |
|  | ANS effectiveness in treating anaphylaxis | 0.812 | 1.000 |
|  | ANS effectiveness compared with AAI | 0.602 | 1.000 |
|  | ANS side effects compared with AAI | 0.056 | 1.000 |
|  | Whether nasal conditions or other nasal medicines affect ANS effectiveness | 0.238 | 1.000 |
|  | ANS expiry date compared with AAI | 0.212 | 1.000 |
|  | ANS cost compared with AAI | 0.152 | 1.000 |
|  | ANS portability or storage compared with AAI | 0.885 | 1.000 |
|  | Environmental impact of ANS compared with AAI | 0.314 | 1.000 |
| **ANS concerns** | Knowing how to use the ANS | 0.865 | 1.000 |
|  | Knowing when to use the ANS | 0.416 | 1.000 |
|  | Whether other people would know when and how to use the ANS | 0.213 | 1.000 |
|  | ANS possibly being painful | 0.307 | 1.000 |
|  | ANS causing other side effects | 0.772 | 1.000 |
|  | ANS not working well enough | 0.326 | 1.000 |
|  | ANS not working as well as AAI | 0.023 | 0.714 |
|  | Being too embarrassed to use the ANS | 0.015 | 0.494 |
|  | Being too scared to use the ANS | 0.129 | 1.000 |
|  | ANS not being easy or practical to carry | 0.299 | 1.000 |
| **Anticipated ANS use and preferences** | Anticipated frequency of carrying the ANS | 0.237 | 1.000 |
|  | Comfort using the ANS rather than an AAI | 0.997 | 1.000 |
|  | Whether device type would affect readiness to use adrenaline | 0.579 | 1.000 |
|  | Prescription preference if offered an ANS | 0.845 | 1.000 |
