# Supplementary material for: International survey of parental and patient perspectives on emerging adrenaline devices for community‐based management of anaphylaxis: An EAACI task force report
Source: Pediatr Allergy Immunol. 2026 Oct 14;37(10):e70507. doi: 10.1111/pai.70507 (PMC13669523; doi:10.1111/pai.70507)
Supplement: Supplementary file 3 [file PAI-37-e70507-s003.zip › Table E9.docx]

**Table E9. Information needs and AAI- and ANS-related concerns according to the child’s age: caregiver survey**

| **Domain** | **Survey item** | **Unadjusted p** | **Holm-adjusted p** |
| --- | --- | --- | --- |
| **AAI concerns** | Knowing how to use the AAI correctly | 0.300 | 1.000 |
|  | Knowing when to use the AAI | 0.891 | 1.000 |
|  | Whether other people would know when and how to use the AAI | 0.750 | 1.000 |
|  | AAI possibly being painful for the child | 0.376 | 1.000 |
|  | AAI causing other side effects | 0.165 | 1.000 |
|  | AAI not working well enough | 0.875 | 1.000 |
|  | Caregiver being too embarrassed to use the AAI | 0.833 | 1.000 |
|  | **Child being too embarrassed to use the AAI** | **<0.001** | **0.003*** |
|  | Caregiver being too scared to use the AAI | 0.010 | 0.315 |
|  | Child being too scared to use the AAI | 0.169 | 1.000 |
|  | AAI not being easy or practical to carry | 0.085 | 1.000 |
| **ANS information needs** | How the ANS works | 0.390 | 1.000 |
|  | Whether the ANS has regulatory approval in the respondent's country | 0.114 | 1.000 |
|  | ANS effectiveness in treating anaphylaxis | 0.168 | 1.000 |
|  | ANS effectiveness compared with AAI | 0.115 | 1.000 |
|  | ANS side effects compared with AAI | 0.120 | 1.000 |
|  | Whether nasal conditions or other nasal medicines affect ANS effectiveness | 0.273 | 1.000 |
|  | ANS expiry date compared with AAI | 0.163 | 1.000 |
|  | ANS cost compared with AAI | 0.218 | 1.000 |
|  | ANS portability or storage compared with AAI | 0.287 | 1.000 |
|  | Environmental impact of ANS compared with AAI | 0.188 | 1.000 |
| **ANS concerns** | Knowing how to use the ANS | 0.552 | 1.000 |
|  | Knowing when to use the ANS | 0.702 | 1.000 |
|  | Whether other people would know when and how to use the ANS | 0.472 | 1.000 |
|  | ANS possibly being painful for the child | 0.566 | 1.000 |
|  | ANS causing other side effects | 0.429 | 1.000 |
|  | ANS not working well enough | 0.194 | 1.000 |
|  | ANS not working as well as AAI | 0.116 | 1.000 |
|  | Caregiver being too embarrassed to use the ANS | 0.661 | 1.000 |
|  | Child being too embarrassed to use the ANS | 0.489 | 1.000 |
|  | Caregiver being too scared to use the ANS | 0.961 | 1.000 |
|  | Child being too scared to use the ANS | 0.314 | 1.000 |
|  | ANS not being easy or practical to carry | 0.216 | 1.000 |
