# Supplementary material for: International survey of parental and patient perspectives on emerging adrenaline devices for community‐based management of anaphylaxis: An EAACI task force report
Source: Pediatr Allergy Immunol. 2026 Oct 14;37(10):e70507. doi: 10.1111/pai.70507 (PMC13669523; doi:10.1111/pai.70507)
Supplement: Supplementary file 3 [file PAI-37-e70507-s003.zip › Tables E1 to E5. Detailed stats.docx]

**Table E1.**

| **Comparison (ANS vs AAI)** | **N** | **W** | **Z** | **p-value** |
| --- | --- | --- | --- | --- |
| Knowing how to use | 461 | 32581.5 | 4.39 | <0.001 |
| Knowing when to use | 474 | 21721.5 | 0.99 | 0.322 |
| Other people knowing how/when to use | 466 | 11483.5 | −6.04 | <0.001 |
| Painful to use | 472 | 11715.5 | −6.13 | <0.001 |
| Other side effects | 464 | 31358.5 | 8.72 | <0.001 |
| Not working well enough | 475 | 27220.5 | 8.83 | <0.001 |
| Parent embarrassment | 484 | 1796.5 | −1.85 | 0.064 |
| Child embarrassment | 462 | 3253.5 | −8.98 | <0.001 |
| Parent too scared | 486 | 4837.5 | −9.92 | <0.001 |
| Child too scared | 457 | 4493.0 | −13.06 | <0.001 |
| Not easy/practical to carry | 477 | 11860.5 | −7.04 | <0.001 |

**Table E2.**

| **Comparison (ANS vs AAI)** | **N** | **W** | **Z** | **p-value** |
| --- | --- | --- | --- | --- |
| Knowing how to use | 200 | 6204.5 | 5.48 | <0.001 |
| Knowing when to use | 153 | 1742.5 | −0.76 | 0.450 |
| Other people knowing how/when to use | 196 | 2973.5 | −2.69 | 0.007 |
| Painful to use | 204 | 860.0 | −4.57 | <0.001 |
| Other side effects | 204 | 4438.0 | 3.58 | <0.001 |
| Not working well enough | 205 | 6249.0 | 6.32 | <0.001 |
| Embarrassment to use | 208 | 810.0 | −3.99 | <0.001 |
| Too scared to use | 206 | 369.5 | −7.10 | <0.001 |
| Not easy/practical to carry | 203 | 2680.0 | −2.28 | 0.023 |

**Table E3.**

| **Item (AAI concern)** | **χ² (df)** | **p-value** | **N** |
| --- | --- | --- | --- |
| Carrying AAI | χ²(4) = 7.47 | 0.113 | 763 |
| Knowing how to use | χ²(4) = 40.65 | <0.001 | 757 |
| Knowing when to use | χ²(4) = 13.45 | 0.009 | 757 |
| Other people knowing how/when to use | χ²(4) = 22.71 | <0.001 | 751 |
| AAI being painful | χ²(4) = 42.41 | <0.001 | 752 |
| Other side effects | χ²(4) = 1.56 | 0.816 | 748 |
| Not working well enough | χ²(4) = 6.57 | 0.160 | 750 |
| Parent embarrassment | χ²(4) = 48.24 | <0.001 | 750 |
| Parent too scared | χ²(4) = 1.60 | 0.809 | 759 |
| Not easy/practical to carry | χ²(4) = 12.92 | 0.012 | 754 |

**Table E4.**

| **Item (ANS concern)** | **χ² (df)** | **p-value** | **N** |
| --- | --- | --- | --- |
| Knowing how to use | χ²(4) = 11.30 | 0.023 | 667 |
| Knowing when to use | χ²(4) = 2.09 | 0.720 | 682 |
| Other people knowing how/when to use | χ²(4) = 10.78 | 0.029 | 680 |
| ANS being painful | χ²(4) = 30.23 | <0.001 | 683 |
| Other side effects | χ²(4) = 18.14 | 0.001 | 678 |
| Not working well enough | χ²(4) = 7.38 | 0.117 | 687 |
| Not working as well as AAI | χ²(4) = 10.55 | 0.032 | 688 |
| Parent embarrassment | χ²(4) = 10.30 | 0.036 | 705 |
| Parent too scared | χ²(4) = 8.53 | 0.074 | 696 |
| Not easy/practical to carry | χ²(4) = 2.73 | 0.603 | 689 |

**Table E5.**

| **Outcome** | **χ² (df)** | **p-value** | **N** |
| --- | --- | --- | --- |
| Comfort using ANS (self) | χ²(4) = 25.39 | <0.001 | 675 |
| Comfort using ANS (child) | χ²(4) = 43.73 | <0.001 | 669 |
| Impact of device type on readiness to use (self) | χ²(4) = 10.42 | 0.034 | 692 |
| Impact of device type on readiness to use (child) | χ²(7) = 675.56 | <0.001 | 687 |
| Prescription preference | χ²(8) = 694.70 | <0.001 | 706 |
