# Supplementary material for: International survey of parental and patient perspectives on emerging adrenaline devices for community‐based management of anaphylaxis: An EAACI task force report
Source: Pediatr Allergy Immunol. 2026 Oct 14;37(10):e70507. doi: 10.1111/pai.70507 (PMC13669523; doi:10.1111/pai.70507)
Supplement: Supplementary file 3 [file PAI-37-e70507-s003.zip › Tables E6 to E8.Thematic analysis ANS.docx]

**Table E6.**

| **Theme** | **Adults: summary and illustrative quotes** | **Caregivers: summary and illustrative quotes** |
| --- | --- | --- |
| Evidence of equivalence and real-world effectiveness (including severe scenarios) | Adults prioritised evidence on whether ANS is as fast, effective and reliable as intramuscular injection, including performance in high‑risk reactions.  • ‘Efficacy compared with intramuscular administration.’  • ‘I would like information on availability, uses, dosage, size, and effectiveness and the differences in the nasal spray and the auto injector, how much longer it takes to be effective… timeline is a big deal.’  • ‘Is it working as quickly as EpiPen or does it take longer to work?’  • ‘ If I’m sick with congested nose will it work?’ | Caregivers’ foremost information need was clinical evidence that ANS works like an AAI in severe, real‑world scenarios (e.g., airway swelling, reduced consciousness). Caregivers emphasised that willingness to use ANS was conditional on this evidence that it is as rapid and effective as an AAI.  • ‘Only concern: that it is as effective as the autoinjector.’  • ‘That it is not effective and that seconds are lost getting to a hospital.’  • ‘Does it work the same way as the adrenaline pen? Is it as fast ... for treating glottic closure? And other symptoms?’ |
| Route-specific nasal factors and tolerability | Participants noted nasal-route factors that could impair delivery (e.g., congestion/blocked nose, nosebleeds) and raised tolerability concerns, including sensitivity to ingredients.  • ‘I have a lot of issues with my nose being blocked most of the time so would never feel comfortable using it’  • ‘being allergic to the ingredients - as the mist is immediately active on nasal passage/trachea’ | Caregivers also highlighted nasal factors (e.g., colds/blocked nose) and wanted reassurance about side effects and tolerability for children.  • ‘What happens if the person has a cold, has the flu? Is it also effective if the nose is blocked?’  • ‘Side effects, or adverse effects in the nose.’ |
| Feasibility under reaction conditions (technique, cooperation, severe symptoms) | Adults worried about whether ANS could be administered correctly during severe reactions (panic, breathlessness), including concerns about dexterity and administering to others.  • ‘I may not be in a position to use it compared to an Epipen when I am starting to go unconscious or trembling during a reaction.’  • ‘Is it easy to use on another person if the person can’t breathe?’ | Caregivers raised practical worries about correct administration in real emergencies, including child cooperation.  • ‘If Nasal spray requires any level of cooperation it may be less effective than epipen (we have had to restrain our child to administer it as he becomes very agitated and confused when has has anaphylaxis.’  • ‘That I apply it correctly.’ |
| Recognition, training, and integration into emergency response | Adults highlighted uncertainty about bystander recognition and perceived seriousness of a spray, and how this might influence emergency response behaviours.  • ‘no one is trained to use a spray, they will not be looking for it. Seems less serious as a spray is used for hayfever, not someone about to die.’  • ‘I would prefer it to an auto injector and I think people would be more likely to help you in an emergency.’  • ‘With the adrenaline injection I know it works: never change a winning team. Also, I wonder whether bystanders would take the reaction seriously if it can be treated with a nasal spray. A needle creates fear and that can work in your favor when you need to get people to call 112.’’ | Caregivers stressed the need for training and clear guidance so schools and other caregivers can recognise when and how to use ANS within existing emergency pathways.  • ‘A new form of treatment—do most people know how to use the spray?’  • ‘Would need to be widely adopted as would want to be public school and friends etc could give if needed [sic]’  • ‘What happens when child is in the care of someone who hasn't been trained with neffy.’ |
| Implementation and unintended use (availability/cost, information gaps, risk of under/over‑use) | Some adults raised practical questions about price, availability, and limited familiarity with ANS, which influenced acceptability.  • ‘Cost and availability. When available.’  • ‘How easy is it to use. How easy is it to "set off" by accident.’  • ‘I have no information.’  • ‘I don't know how it works.’ | A minority of caregiver responses also noted that if ANS feels easier/less frightening, it could change thresholds for use, highlighting the importance of clear indications and education.  • ‘The hesitation/inhibition to try it without an allergic reaction could be lower.’  • ‘I think this is less frightening than the injection.’ |

**Table E7.**

| **Theme** | **Adults: summary and illustrative quotes** | **Caregivers: summary and illustrative quotes** |
| --- | --- | --- |
| Evidence of equivalence and real-world effectiveness (including severe scenarios) | Adults wanted evidence comparing ANS with intramuscular injection and clarity on effectiveness in severe presentations (e.g., airway compromise).  • ‘Efficacy compared with intramuscular administration.’  • ‘...how much longer it takes to be effective… time line is a big deal.’  • ‘My airways close with anaphylaxis - is the spray effective in this case?’ | Caregivers asked for real-world evidence, including scale of testing and effectiveness in severe scenarios such as reduced consciousness.  • ‘How many people it’s been tested on and its efficacy’  • ‘Substantial evidence of efficacy’  • ‘I would like to know about it's effectiveness if a child has lost consciousness…’ |
| Dosing, eligibility, and number of doses | Adults asked about dose adequacy, whether multiple doses are possible, and whether effectiveness varies by weight.  •’ how many doses are contained in the spray...single or multiple uses.’  • ‘Impact on people with larger weight.’ | Caregivers emphasised eligibility (including weight/age thresholds), dosing and whether one or more doses are needed, particularly for children.  • ‘Hope it will soon be available for children under 30 kg.’  • ‘Does it have to be adjusted according to my child's weight, or is there one for adults and a version for children?’  • ‘Does the spray definitely deliver a sufficient dose.’ |
| Safety profile, ingredients, and contraindications | Adults wanted detail on active/inactive ingredients and contraindications, including concerns about excipients and allergies.  • ‘The ingredients both active and inactive.’  • ‘Ingredients - I know autoinjectors have sulfite (specialist says it better to use than not use injector). However if the nasal spray had such I would be more worried as this would enter my trachea and could be more dangerous.’  • ‘What the ingredients are as I also have life threatening allergy to polyethylene glycol.’ | Caregivers sought reassurance about side effects and contraindications, especially in paediatric use.  • ‘Guarantee of effectiveness and what the side effects are.’  • ‘Whether it will already be available for very young children and whether having a nose full of mucus would reduce the effectiveness of the spray.  • ‘Efficacy and long term use side effects vs adrenaline.’ |
| Practicalities: storage, shelf-life, and device design | Practical questions included storage requirements (heat/cold), device handling, and other design considerations.  • ‘how many doses are contained in the spray...single or multiple uses.’  • ‘The countries using it now.’  • ‘Stable in heat/cold?’ | Caregivers asked about storage temperature, shelf-life, and device robustness/packaging to support day‑to‑day carriage.  • ‘Storage temperature?’  • ‘Expiry dates!’  • ‘It would be much more comfortable to carry and store, especially in summer.’ |
| Integration into action plans and post-use care | Adults asked how ANS fits within emergency action plans (e.g., whether to call an ambulance and whether it is single-use or can be combined with an autoinjector).  • ‘Do you also have to call an ambulance/emergency doctor after using it?’  • ‘Would it be single use?’ | Caregivers similarly sought clarity on when to use ANS, when to seek emergency care afterwards, and how it aligns with existing plans.  • ‘Does it also require immediate travel to the hospital, as currently happens with the injector?’ |
| Access, approval, implementation, and recognisability (including confusion risk) | Adults requested information on availability by country and emphasised that ANS should be clearly recognisable to avoid confusion with other nasal sprays.  • ‘The countries using it now.’  • ‘How to store it, if can be combined with EpiPen if a second dose is needed, how is the packaging I.e does it look like a nasal spray for the flu? Dont want anyone to use it by mistake, need to look very different from common nasal sprays.’  • ‘I am afraid people will mistake the nasal epinephrine for Naloxone and not administer it when needed.’ | Caregivers asked when and where ANS would be available, whether it would be prescribed/funded, and how it would be implemented (including in schools).  • ‘I would like to know if here in Spain it is already available’  • ‘When will it be available in Australia.’  • ‘whether it can be distributed in schools on request, as the injector is or should be.’  • ‘It must be ensured, like the intramuscular one, that it is always available and that it is funded.’ |

**Table E8.**

| **Theme** | **Adults: summary and illustrative quotes** | **Caregivers: summary and illustrative quotes** |
| --- | --- | --- |
| Practical burdens: carriage, storage, expiry and availability | Adults described burdens of carrying AAIs (size/portability) and managing storage conditions and expiry, including concerns about pharmacy stock.  • ‘I find storage difficult (temperature, size, etc.).’  • ‘The biggest issue is the size of injectors and the temperature restrictions.’  • ‘Price, short expiry, storage conditions, size.’ | Caregivers similarly highlighted the day‑to‑day burden of having AAIs always available and stored correctly.  • ‘The temperature is a nuisance.’  • ‘Keeping it at the right temperature in rural Australia where the temps get high.’ |
| Needle fear, pain, and anxiety about injection effects | Adults reported anxiety about injecting (pain) and worries about bodily effects/side effects after use.  • ‘Not knowing what it does to my body, what it feels like, and how I know whether it works or doesn't.’  • ‘Someone told me you can hit the thigh bone, and for some reason that just freaks me out...’  • ‘Immediate side effects and lack of timely medical help.’ | Caregivers described needle fear, panic and hesitation at the moment of administration.  • ‘The device is frightening; I'm afraid of doing it wrong, I don't know ... it freezes/paralyzes me, and I'm extremely afraid something will happen to him.’  • ‘That he can't use it; too great an inhibition because of the needle.’  • ‘Being so nervous and freezing at the moment of administering the autoinjector.’ |
| Decision-making under uncertainty and post-use pathway burdens | Adults worried about judging when to use an AAI and the consequences afterwards (e.g., calling emergency services, hospital attendance).  • ‘Difficulty deciding when it's the right moment to use it; not knowing well the reactions after an injection so as to understand they shouldn't be frightening.’  • ‘If I used the injector, according to the leaflet I'd have to call an ambulance/emergency doctor afterwards. I'd like to avoid that.’  • ‘Hospital stay for at least 48 hours after use; that I won't use it in time; that I won't press hard enough so it doesn't trigger.’ | Caregivers frequently expressed uncertainty about when a reaction is severe enough to warrant use, contributing to delayed or hesitant administration.  • ‘We worry about administering the autoinjector when it isn't a severe reaction, and how to identify when it should be given.’  • ‘It is not really clear when it should be used. Sometimes I’m told it’s only for this exact symptoms on the action plan, but most medical practitioners, in hospitals especially, use the guide that if two or more parts of the body are reacting then give it. It is very stressful working out where that line is to use it, working out if it’s manageable and not wanting to overuse/ overreact to make your child fearful. There is such a massive grey zone around when to use the pen that it is actually very stressful to manage.’ |
| Reliability and confidence in others’ ability to administer | Adults raised concerns about device reliability and whether others would be able to administer correctly in an emergency.  • ‘Only that someone else will do it wrong.’  • ‘Malfunction.’  • ‘Lack of education in society on the purpose and use of epinephrine injectors.’ | Caregivers emphasised reliance on schools/other adults and worries about others’ competence in recognising anaphylaxis and using the device.  • ‘Whether other adults caring for him would be able to identify anaphylaxis and be confident to administer it.’  • ‘Fear that the team at school and in other activities won't know how and when to act, despite information and training.’  • ‘Cannot leave child with any other person, unless epipen trained. Cannot leave at parties, or activities. Difficult to use holiday clubs or casual childcare. It's very limiting.’ |
| Managing severe reactions and aftercare logistics (multiple doses, delayed medical help) | Adults worried about severe reactions requiring multiple doses and the risk of being far from timely medical help.  • ‘That a single dose will not be enough if I'm in a situation where additional medical support may not be timely.’  • ‘Whether the first 2 will fail and we will need another before an ambulance arrives.’  • ‘That I will wait until it is too late to give myself, also that I will not have enough Medicne to stop my reaction as I have severe reactions.’ | Caregivers also noted system-level barriers and aftercare logistics, including access to replacement devices and emergency pathways.  • ‘Trying to get them to the ER afterward, other caretakers knowing when to use it and actually doing it right, the price of EpiPen.’  • ‘Having to reach a hospital as quickly as possible after administering the EpiPen.’  • ‘Unfortunately the autoinjector is not available to buy in our country. We tried to buy it during a trip to the USA, but we were not able.’ |
| Social restrictions, stigma, and peer pressure | Not a prominent theme in adult responses to this question. | Caregivers described how allergies and carrying AAIs can affect social participation, stigma and peer dynamics.  • ‘No family invites him to visit their homes because the burden of the autoinjector is very heavy and it creates even more fear for those who have never dealt with allergy.’  • ‘As a primary school student, I am not too worried (about my child) as auto injectors are managed by the school staff and lunches are packed. In a couple of years when he starts high school he is responsible for his own autoinjector and lunch and peer pressure from friends.’  • ‘Teenage life- relationships, kissing and partners knowing safety and when to use auto injector.’ |
